# Optimizing maize-soybean intercropping patterns under film-edge cultivation regulates soil bacterial communities to enhance productivity and water use efficiency

**DOI:** 10.3389/fpls.2025.1683061

**Published:** 2025-11-03

**Authors:** Jingyi Hu, Jiaxin Wang, Panpan Zhang, Lixin Tian, Yuhao Yuan, Qian Wei, Tao Wang, Yan Luo, Yaning Guo, Xiaolin Wang, Xiong Zhang

**Affiliations:** ^1^ Modern College of Agriculture/Dryland Agricultural Engineering Technology Research Center in Northern of Shannxi, Yulin University, Yulin, China; ^2^ School of Agriculture, Anhui Agriculture University, Hefei, China; ^3^ School of Agriculture, Henan Agriculture University, Zhengzhou, China

**Keywords:** maize-soybean intercropping, bacterial diversity, soil physicochemical properties, yield, water use efficiency

## Abstract

Intercropping enhances resource utilization and productivity through interspecific complementarity; however, its potential in arid-region film-edge cultivation, especially microbial-environmental interactions, remains underexplored. A two-year field experiment was conducted in film-edge cultivation in northern Shaanxi, comparing maize monoculture (CJCJ), soybean monoculture (SJSJ), and four maize–soybean intercropping patterns—2C2S, 2C4S, 4C2S, and 4C4S—to investigate how these configurations affect soil bacterial community composition and crop productivity. Results showed that the 4C2S and 2C4S patterns improved soil moisture dynamics, enzyme activities, and microbial community structure. Compared with monocropping, intercropping increased soil moisture in both crop zones, with differential regulation. In 2022, all intercropping treatments significantly increased maize rhizospheric urease, alkaline phosphatase, and sucrase activities (*P* < 0.05). In 2023, 2C4S significantly enhanced these three enzymes plus catalase in maize vs. CJCJ (*P* < 0.05), while 4C4S significantly increased soybean catalase and sucrase (2022–2023) and urease and alkaline phosphatase (2023) vs. SJSJ (*P* < 0.05). Intercropping improved the rhizospheric root morphology of crop. 2022 2C4S significantly increased soybean root length and average diameter at R1 stage (*P* < 0.05); 2023 2C4S significantly enhanced maize root dry weight, length, surface area, and volume at V12 stage vs. CJCJ (*P* < 0.05). Maize bacterial richness was unaffected, but 2C2S and 4C2S significantly increased soybean bacterial richness and Shannon diversity (*P* < 0.05). At maize V12, 2C4S and 4C4S increased *Sponge phylum* by 24.68% and 32.85%, and 2C4S increased *Dentomonas* by 22.92% vs. monocropping. At soybean R1, 2C4S increased *Sponge* by 7.34%, 2C4S and 4C2S increased *Acidobacteria* by 15.64% and 21.40%; 2C4S enriched *Nitrososphaeria_A* and *Blastocatellia*, 2C2S was dominated by *Bacteroidia*, and 4C4S had the highest total soybean microbiota abundance. The treatment-year interaction significantly affected maize yield and WUE (*P* < 0.05), and had a highly significant effect on those of soybean (*P* < 0.01). 2C4S significantly boosted maize yield (2022: 4.04%-33.53%; 2023: 2.58%-26.98%) and exhibited the least soybean yield reduction under intercropping compared to SJSJ. In conclusion, by regulating the soil-microbe-crop nexus, 2C4S increases maize yield, reduces soybean loss, and improves resource efficiency in arid film-edge cultivation, providing a viable strategy for intercropping in water-limited regions.

## 1 Introduction

Amid the global transition toward low-carbon, sustainable agriculture, clean production has emerged as a priority, aiming to minimize non-point source pollution through efficient resource utilization and eco-friendly technologies (Hussain et al., 2025). Maize–soybean intercropping, as a representative ecological planting model, enhances the use of light, heat, water, and nutrients through interspecific complementarity (Xu et al., 2020). This approach reduces reliance on chemical fertilizers and pesticides, achieving simultaneous yield gains and improvements in soil fertility, thus aligning with clean production goals (Nasar et al., 2024; Xia et al., 2024). Prior research confirms the system’s positive impacts on nutrient cycling, microbial activity, and crop productivity (Smith et al., 2016; Chen et al., 2025). However, in arid and semi-arid regions, where water scarcity and extensive cultivation dominate, the co-occurrence of yield instability and environmental degradation poses a major challenge (Hou et al., 2024; Ren et al., 2024). While the synergy between intercropping and clean production is promising, the integration of water-saving technologies into this system remains insufficiently studied, particularly concerning how to balance water-use efficiency, soil health, and pollution mitigation.

In dryland agriculture, the film-edge cultivation method (i.e., precision planting 5–7 cm away from the side of the plastic film)—also known as ridge-film and furrow-planting—has emerged as a viable alternative to full-film mulching. In this system, plastic films cover ridges harvest rainwater, while crops are sown in the furrows. This technique not only enhances precipitation use by channeling rainwater into planting zones but also alleviates salt accumulation and aeration issues associated with continuous full-film coverage. Moreover, its closed planting layout improves film recovery rates and reduces residual pollution, thereby supporting cleaner production systems (Chang et al., 2024).

Existing monocropping studies have demonstrated that film-edge cultivation maintains or even enhances maize yields compared to traditional mulching, while reducing labor input and residual plastic pollution (Zhang B, et al., 2023). Additionally, mulching can stabilize soil moisture, improve nitrogen-fixing microbial communities, and enhance soybean yields (Liao et al., 2022; Farmer et al., 2017). Under bare-soil conditions, intercropping systems have already been shown to influence soil microbial diversity and enzymatic activities significantly (Xiao et al., 2023; Dang et al., 2020). Compared with the monocropping system, the maize-soybean intercropping system significantly enhances the diversity and functional traits of rhizosphere microbial communities while increasing the relative abundance of key genes involved in soil nitrogen cycling, thereby sustaining the crop yield advantage (Zhang S, et al., 2023; Shu et al., 2024). Additionally, maize-legume intercropping promotes crop growth and soil nutrient cycling by modulating soil enzyme activities (Liu et al., 2024a). For instance, Lei et al (2023) reported elevated catalase in maize and urease in soybean under the same 2:2 arrangement. However, most existing studies have focused on monoculture systems or bare-soil intercropping, leaving a critical gap regarding how intercropping patterns interact with film-edge cultivation. Although this study focuses on soil nutrients and microorganisms, the occurrence of pests and diseases in two crops under intercropping mode and their prevention and control strategies are equally crucial. Two key knowledge gaps remain: First, the synergistic interactions between the unique microenvironment created by film-edge cultivation and the complementarity in intercropping have not been fully elucidated, particularly their effects on soil physicochemical properties, microbial community structures, and yield formation. Second, the compatibility between various intercropping patterns and film-edge cultivation remains largely unexamined, limiting our ability to harness the full ecological and yield-enhancing potential of these integrated systems in arid regions.

Northern Shaanxi, a representative arid and semi-arid area, relies heavily on maize and soybeans as staple crops. Persistent water shortages and traditional planting practices have resulted in unstable yields and soil degradation. Given the high interannual climate variability in semi-arid northern regions, single-year experiment data are prone to interference from accidental climate events. This study was therefore conducted consecutively in 2022–2023 to reduce climatic heterogeneity impacts, verify the stability of intercropping effects, enhance conclusion reliability. To address these limitations, this study evaluates how different maize–soybean intercropping patterns under film-edge cultivation affect soil properties, microbial diversity, and crop yields in this region. The outcomes provide a scientific foundation for developing high-yield, environmentally sustainable maize–soybean cultivation systems in northern Shaanxi, contributing to the broader goal of modern agricultural sustainability.

## 2 Materials and methods

### 2.1 Field site, and experiment design

**Figure 1:**
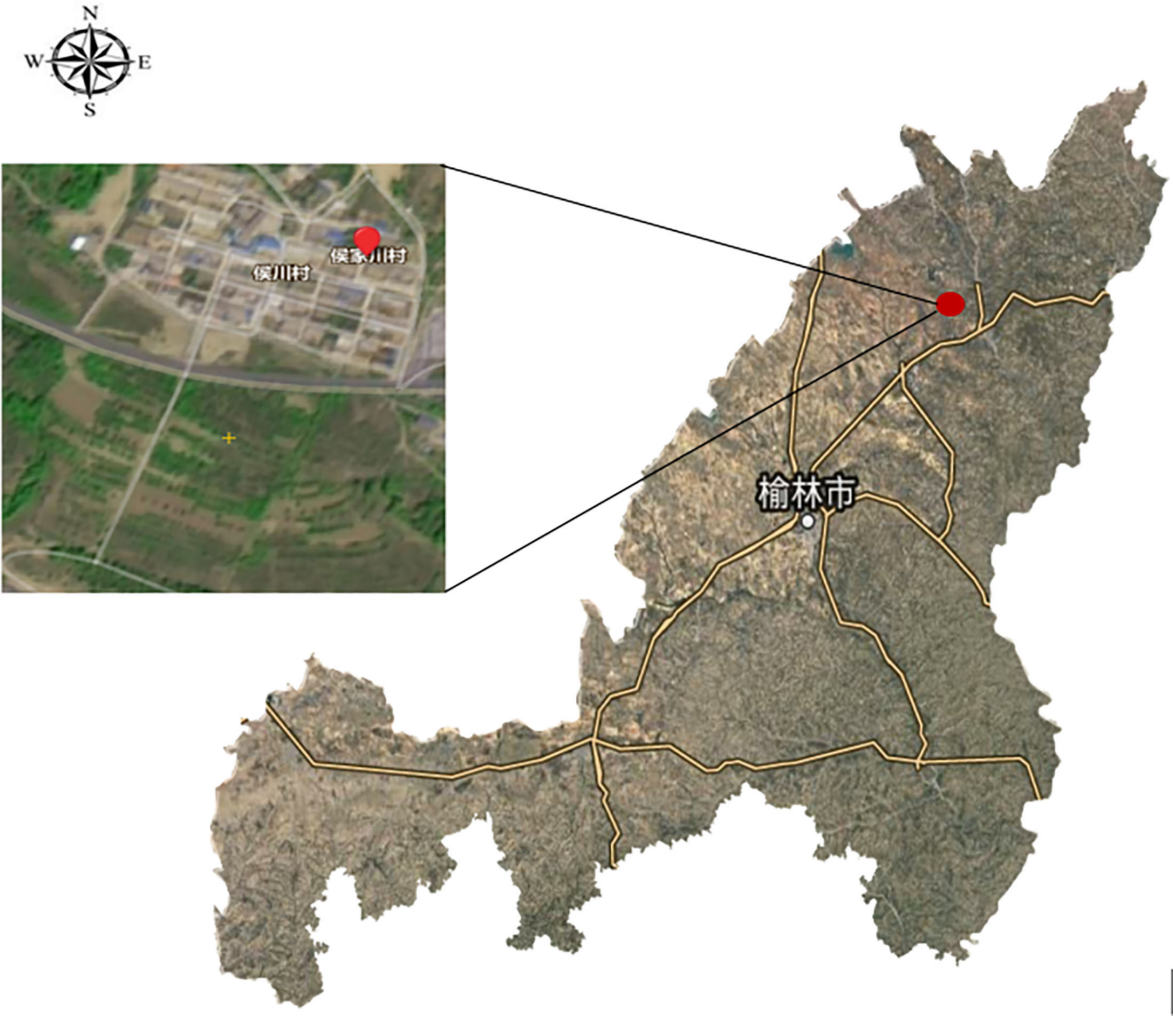
Overview of the study area.

The field experiment was conducted from May to October in both 2022 and 2023 at the Agricultural Technology Extension Center Experimental Station in Shenmu City, Shaanxi Province (**Figure 1**). The site is located in Houjiachuan Village, Shenmu County (110°30´E, 38°48´N). During the off-cropping seasons, experimental plots were kept fallow with no crop cultivation to avoid interference. To ensure uniform initial experimental conditions and minimize the impact of soil background variability on results, a comprehensive baseline soil survey was performed on each plot via the five-point sampling method before the experiment commenced. Specifically, the soil at the experimental site is characterized by an organic matter content of 10.85 g·kg^-^¹, total nitrogen of 0.56 g·kg^-^¹, available phosphorus of 10.95 mg·kg^-^¹, and available potassium of 107 mg·kg^-^¹. For these properties, soil organic matter content was determined via the potassium dichromate oxidation-external heating method, total nitrogen via the Kjeldahl method, available phosphorus via the sodium bicarbonate extraction-molybdenum antimony anti-colorimetric method (Olsen method), and available potassium via the ammonium acetate extraction-flame photometric method.

**Figure 2:**
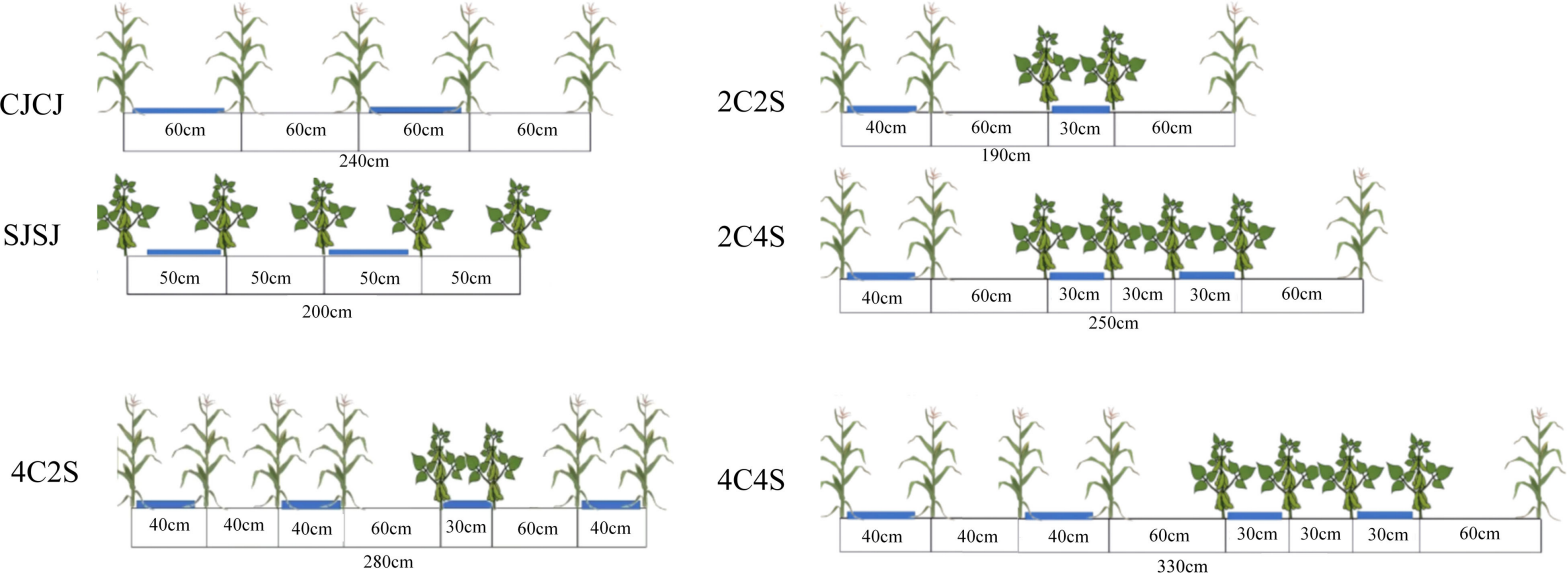
Planting pattern diagram of maize-soybean intercropping.

The maize variety used was Xianyu 335, and the soybean variety was Zhonghuang 30. In this study, a random design was adopted. Six treatments were established, all based on film-edge cultivation maize monoculture (CJCJ), soybean monoculture (SJSJ), and four maize-soybean intercropping systems—2C2S (2 maize:2 soybean), 2C4S (2:4), 4C2S (4:2), and 4C4S (4:4) (**Figure 2**, **Supplementary Table S1**). Monoculture plots measured 7.5 m × 5 m (37.5 m²), while intercropping plots contained three belts per plot, each 5 m long. Each treatment was replicated three times. In 2022, the planting densities of maize and soybean in the experiment were set at 50,000 plants·ha^-1^ and 120,000 plants·ha^-1^, respectively. Based on the experimental results obtained in 2022, the planting densities and treatment schemes were optimized in 2023: specifically, the maize planting density was adjusted to 60,000 plants·ha^-1^, while the soybean planting density was adjusted to 90,000 plants·ha^-1^. Furthermore, in accordance with China’s national technical guidelines for soybean-maize strip intercropping, the planting densities of maize and soybean under the strip intercropping system were comparable to those under their respective monocropping systems.

In 2022, sowing of maize and soybean was conducted on May 4th, with soybean harvested on September 15th and maize harvested on October 1st; in 2023, sowing of maize and soybean was carried out on May 6th, and both crops were harvested on October 1st. Before sowing, base fertilizer was applied by broadcasting 300 kg·ha^-1^ of diammonium phosphate for maize and 225 kg·ha^-1^ of compound fertilizer (15-15-15, referring to N-P_2_O_5_-K_2_O) for soybean. Meanwhile, drip irrigation tapes were installed, and 0.01-mm-thick white conventional plastic mulch with a light transmittance and thermal radiation rate exceeding 90% was applied, while tailored management practices were implemented for different cultivation patterns: specifically, for film-edge cultivation, flat low ridges were established, seeds were sown 3–5 cm alongside the mulch, and the mulch width was determined by the crop row spacing. During the maize jointing stage, 300 kg·ha^-1^ of urea was broadcast as topdressing, and all experiments followed the requirements of the Chinese Regional Trial for Maize and Soybean Varieties to perform intertillage, weeding, and control of diseases, pests, and weeds.

Drip irrigation under plastic mulch was adopted in this experiment, using 16 mm in-line labyrinth drip tape with a designed flow rate of approximately 0.01 m/s. The irrigation amount was dynamically regulated according to soil moisture content: irrigation was initiated when the soil moisture content in the 0–20 cm soil layer decreased to 60% of field capacity, and ceased when the field capacity of this soil layer rose back to 70%.The total irrigation amount during the 2022 experimental period was 0 m^3^·ha^-1^ (no artificial irrigation was conducted); during the 2023 growing season (May to October), 2 rounds of artificial irrigation were implemented, with a cumulative irrigation amount of 540 m^3^·ha^-1^. Importantly, the irrigation management measures were completely consistent across all intercropping treatments and monocropping treatments. This consistency was intended to avoid interference with the comparative analysis of soil hydrothermal environments and microbial communities among different treatments due to differences in irrigation.

### 2.2 Sample collection and measurements

In 2022, maize was sampled at 32, 46, 76, 124, and 150 days after sowing (DAS), corresponding to the vegetative 6th leaf (V6), vegetative 12th leaf (V12), silking (R1), filling (R4), physiological maturity (R6) stages, respectively; soybean was sampled at 32, 46, 76, 124, and 134 DAS, corresponding to the first trifoliolate (V1), beginning flowering (R1), peak pod (R4), full seed (R6), full maturity (R8) stages, respectively.In 2023, both maize and soybean were sampled at 33, 58, 89, 127, and 148 DAS.

At the key growth stages of maize and soybean, three plants with uniform growth vigor were randomly selected for each crop under each treatment. The square excavation method was adopted: with the plant as the center, an excavation range with a radius of approximately 20 cm was delimited, and the intact root system was carefully excavated. A soft-bristled brush was then used to carefully collect the rhizosphere soil adhering to the root surface. The collected rhizosphere soil was divided into two portions for subsequent processing: one portion was air-dried naturally and used for the determination of soil physicochemical properties, while the other portion was kept in a fresh state, immediately stored at -80°C for cryopreservation, and later sent to a professional testing company for microbial index analysis.

The excavated intact root systems were placed into 1-mm mesh bags and gently rinsed with low-pressure running water to remove residual soil. After cleaning, a root scanner was used to scan and analyze the root morphology. Subsequent to the morphological analysis, the root samples, together with the preprocessed aboveground parts (stems, leaves, and ears), were first oven-dried at 105°C for 30 minutes to deactivate enzymes, and then transferred to a constant-temperature oven at 80°C for drying to a constant weight. These samples were ultimately used to determine the dry matter accumulation of each plant organ.

When maize or soybean was arranged in 4 rows in the intercropping system, row-specific sampling was conducted to eliminate the interference of edge effects on the determination results: for the target crop, three sampling points were randomly selected for each of the two row positions (inner two rows and outer two rows), resulting in a total of 6 sets of determination data. By covering samples from different row positions, the representativeness of the results was ensured.This sampling approach was uniformly applied to the collection of samples for all determination indicators in this study, covering root samples, soil samples, and samples related to seed testing and yield measurement, thereby ensuring both the consistency of sampling methods across all indicators and the representativeness of the data.

#### 2.2.1 Soil temperature and soil water

Soil temperature at depths of 0, 5, 10, 15, 20 and 25 cm in the root zone was recorded during the maize R4 (filling stage) and soybean R6 (full seed stage) using a right-angle soil thermometer, with measurements taken between 10:00 and 12:00 a.m. Soil moisture was assessed in both wide and narrow rows using the gravimetric method at 0–100 cm depth, sampled at 20 cm intervals. Soil samples were promptly sealed in aluminum boxes, and moisture content was calculated based on weight loss after oven drying.

#### 2.2.2 Determination of soil physical and chemical properties

Rhizosphere soil was collected during the maize V12 and soybean R1 stages. Soil pH was measured following the National Environmental Protection Standards of the People’s Republic of China using a 1:2.5 (m/V) soil-to-distilled water ratio. After 30 minutes of shaking, pH was recorded using an FE28-pH meter.

Soil enzyme activities were analyzed as follows: urease (URE) using the phenol-sodium hypochlorite colorimetric method, alkaline phosphatase (ALP) by sodium phosphate colorimetry, catalase (CAT) by potassium permanganate titration, and sucrase (SUC) via 3,5-dinitrosalicylic acid colorimetry.

#### 2.2.3 Determination of soil microorganisms

In 2023, microbial analyses were performed on the rhizosphere soil of maize at the V12 and soybean at the R1. Microbial indicators included α-diversity indices (ACE, Chao1, Shannon, and Simpson), β-diversity, and microbial community composition. Operational taxonomic units (OTUs) were calculated to quantify shared and unique taxa across samples based on relative abundance tables. The original data comes from Wekemo Bioincloud. The raw data were deposited in CNGB-NGDC GSA under the accession number subCRA026869.

#### 2.2.4 Crop root morphology

At the V6 and V12 stages of maize, and the V1 and R1 stages of soybean, root samples were scanned individually using an Epson Perfection V800 Photo scanner after cleaning. Root morphological parameters, including total root length, surface area, and volume, were analyzed using WinRHIZO software.

#### 2.2.5 Production and constituent factors

For yield and yield component analysis, samples were collected after full maturity of maize and soybean. In each treatment, three sampling points were selected. At each point, ten consecutive maize ears from a single row and ten soybean plants from a single row were harvested for seed trait evaluation. Additionally, all maize and soybean plants within a 3-meter row length at each sampling point were harvested to determine plot-level yield. From the air-dried grains of each treatment, three 100-seed subsamples were randomly selected and weighed individually. The average value was calculated as the 100-seed weight for maize and soybean in each treatment.


$$Y=\frac{Y_{M}}{A_{M}}\times 10,000\times R_{land}\times \frac{1-M_{A}}{1-M_{S}}$$

Y: theoretics maize or soybean grain yield, (kg ha^-1^); ${\text{Y}}_{A}$ :measured maize or soybean grain yield from sampling plots (kg); ${\text{A}}_{A}$ : area of maize or soybean sampling plots (m^2^); 10,000: Conversion factor for area from m^2^ to ha; ${\text{R}}_{land}$ : Land occupancy rate of maize or soybean (e.g., under the 2C2S intercropping pattern, the land occupancy rate of maize was 10/19, while that of soybean was 9/19.); ${\text{M}}_{A}$ : actual moisture content of maize or soybean grains; ${\text{M}}_{S}$ : standard Moisture Content (SMC, fixed value). The SMC for maize was 14%, and that for soybean was 13.5%.

## 3 Results

### 3.1 Effects of different intercropping patterns of maize and soybean on soil water temperature status

#### 3.1.1 Effects of different intercropping patterns of maize and soybean on soil moisture content

**Figure 3:**
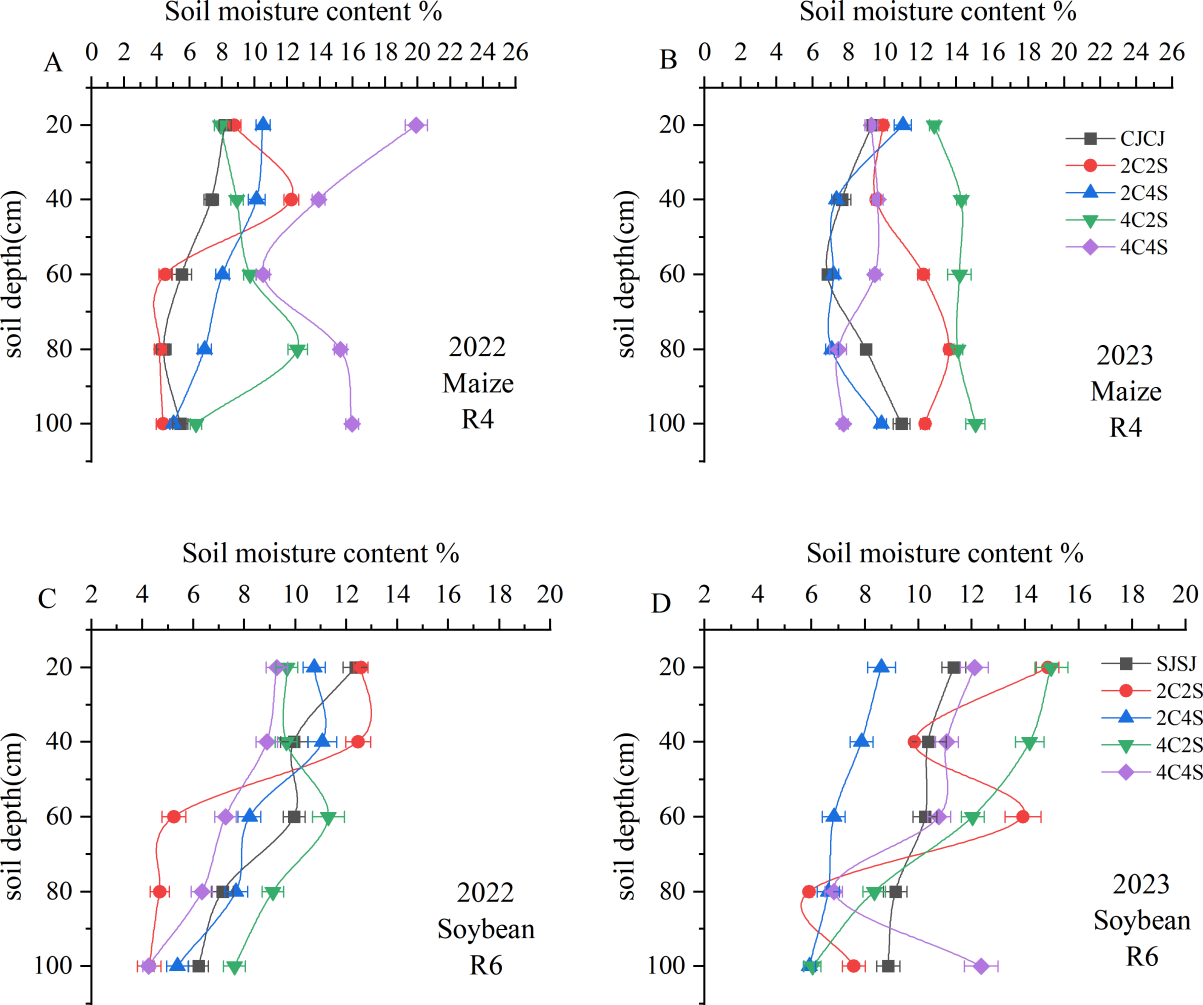
Effects of different intercropping patterns on soil moisture content in maize and soybean. **(A)** Soil moisture content during the maize R4 period in 2022. **(B)** Soil moisture content during the maize R4 period in 2023. **(C)** Soil moisture content during soybean R6 period in 2022. **(D)** Soil moisture content during soybean R6 period in 2023.

During maize R4 stage in 2022, the 4C4S intercropping system maintained significantly greater soil water content across the 0–100 cm profile compared to other treatments, with the exception of the 60 cm layer (*P* < 0.05); this represented a 45%-56.84% increase relative to the CJCJ (**Figure 3A**). In 2023, both 2C2S and 4C2S intercropping regimes exhibited significantly higher soil moisture in the 0–100 cm layer than CJCJ (*P* < 0.05). In particular, 4C2S notably enhanced water content in the upper-middle layers (0–60 cm) of maize rows (*P* < 0.05) (**Figure 3B**).

Conversely, in soybean-growing zones, soil moisture followed a “decrease-then-increase” pattern, with the lowest levels observed in the 0–40 cm layer (**Figures 3C, D**). At soybean R6 stage in 2022, 2C2S ranked the highest in soil water content within the 20–40 cm layer (*P* < 0.05), while 4C2S outperformed all other intercropping and monoculture systems in the 60–100 cm layer (*P* < 0.05). During soybean R6 stage in 2023, soil moisture under 2C2S fluctuated sharply in the 40–80 cm layer, with the 60 cm depth showing significantly higher levels than other treatments (*P* < 0.05). Of note, in the 0–60 cm range, 4C2S had significantly higher moisture than the SJSJ monoculture (*P* < 0.05), whereas 4C4S showed a numerical increase over SJSJ without statistical significance (*P* > 0.05). This underscores the divergent impacts of varying intercropping patterns on soil water retention in soybean fields.

#### 3.1.2 Effects of different maize-soybean intercropping patterns on soil temperature

**Figure 4:**
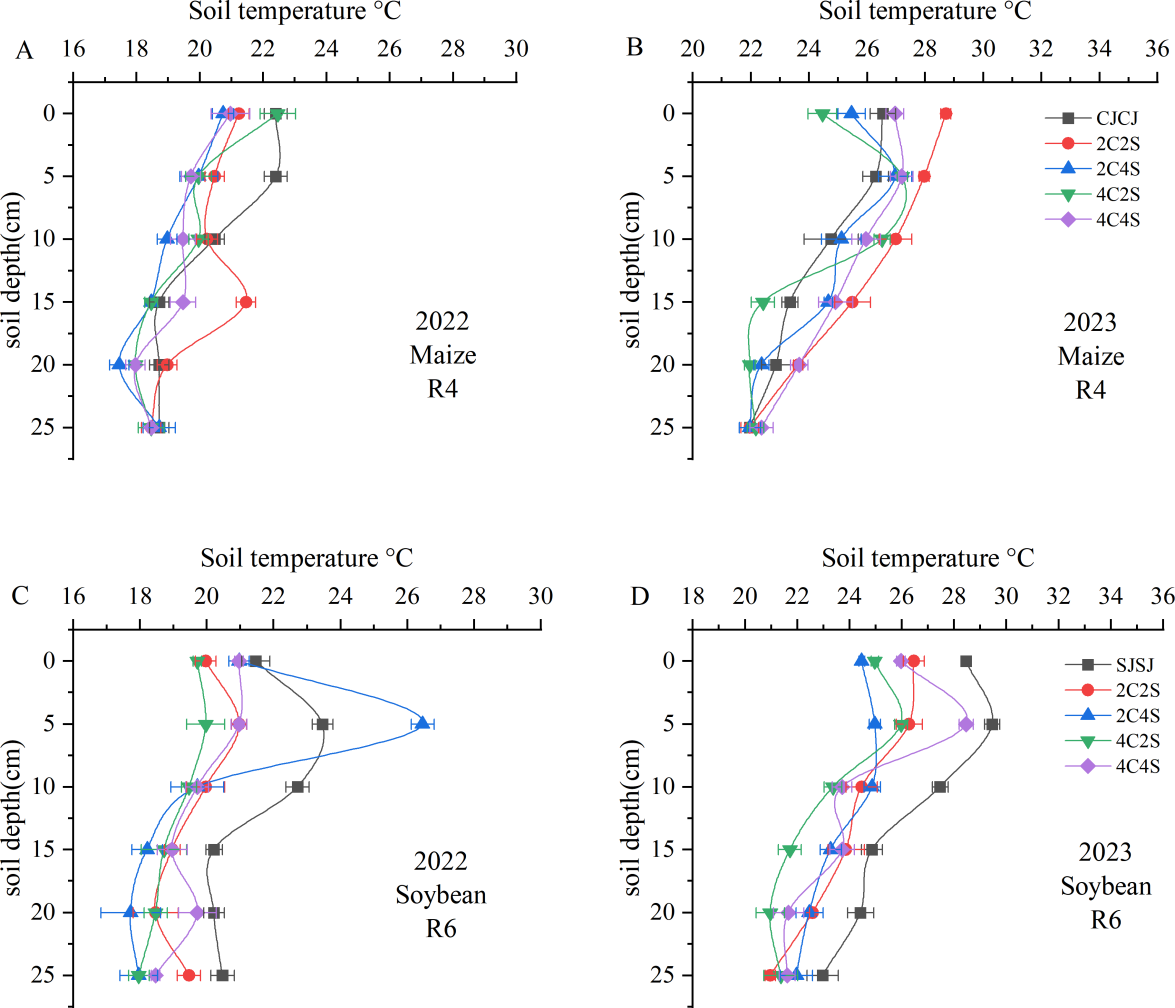
Effects of different intercropping patterns on soil temperature in maize-soybean systems. **(A)** Soil temperature during the maize R4 period in 2022; **(B)** Soil temperature during the maize R4 period in 2023; **(C)** Soil temperature during the soybean R6 period in 2022; **(D)** Soil temperature during the soybean R6 period in 2023.

Comparisons between 2022 and 2023 revealed that soil temperature generally decreased with increasing soil depth, and differences among treatments diminished as depth increased (**Figure 4**). Data from the maize R4 stage in 2022 showed that the mean soil temperature in the 0–10 cm layer under the CJCJ monocropping system was significantly higher than that under all intercropping systems (*P* < 0.05); however, in the 10–20 cm layer, the 2C2S treatment exhibited a significantly higher mean temperature than the CJCJ monocropping (*P* < 0.05). Monitoring results in 2023 indicated that within the 0–20 cm layer, the mean soil temperatures under the 2C2S and 4C4S treatments were significantly increased by 4.6%-10.17% and 1.47%-6.20%, respectively, compared with the CJCJ monocropping (*P* < 0.05).

A similar pattern was observed in the soybean system: soil temperature declined as depth increased, with treatment differences gradually narrowing (**Figures 4C, D**). During the soybean R6 stage in 2022, the 2C4S treatment significantly increased the 5 cm soil temperature by 2.32%-11.32% relative to other treatments. Additionally, the SJSJ monocropping had significantly higher temperatures in the 10–25 cm layer than other treatments (*P* < 0.05), except for the 20 cm layer—where its temperature was higher than that of the 4C4S treatment but without statistical significance (*P* > 0.05). In contrast, during the soybean R6 stage in 2023, the SJSJ monocropping showed higher temperatures in the 15 cm layer than the 2C2S treatment and in the 25 cm layer than the 4C4S treatment, but neither difference was significant (*P* > 0.05). Beyond these two depths, the SJSJ monocropping had significantly higher soil temperatures than all intercropping treatments across the remaining depths in the 0–25 cm layer (*P* < 0.05). These results highlight the interannual variability in the effect of intercropping on soil thermal dynamics.

### 3.2 Effects of different maize-soybean intercropping patterns on physical and chemical properties of rhizosphere soil

In **Table 1**, for the maize system in 2022, the soil pH of all intercropping treatments was significantly higher than that of CJCJ (*P* < 0.05), while no significant differences were observed among intercropping treatments (*P* > 0.05). Soil enzyme activities exhibited a positive response to intercropping: the activities of soil urease, alkaline phosphatase, and sucrase in all intercropping treatments were significantly higher than those in the CJCJ monoculture (*P* < 0.05). However, except that the catalase activity in intercropping treatments was higher than that in the 2C2S treatment without statistical significance (*P* > 0.05), the catalase activity of other intercropping treatments was significantly lower than that of the CJCJ treatment (*P* < 0.05). Among these, the activities of alkaline phosphatase and sucrase under the 4C2S and 4C4S treatments were significantly enhanced (*P* < 0.05), increasing by 78.78%, 69.69% and 495.24%, 523.81% compared with CJCJ, respectively. This indicates that intercropping can improve soil pH and most soil enzyme activities in the maize system.

**Table 1:** Effects of different maize-soybean intercropping patterns on soil physicochemical properties.

Year	Crop	Treatments	pH	Urease activity (U·g^-1^)	Alkaline phosphatase activity (U·g^-1^)	Catalase activity (U·g^-1^)	Sucrase activity (U·g^-1^)
2022	Maize	CJCJ	8.66 ± 0.01b	7.83 ± 0.42d	0.33 ± 0.01c	9.39 ± 0.74a	0.21 ± 0.01d
		2C2S	8.82 ± 0.12a	12.56 ± 0.76a	0.48 ± 0.02b	9.05 ± 1.45ab	0.49 ± 0.04c
		2C4S	8.89 ± 0.11a	8.45 ± 0.16b	0.32 ± 0.004c	8.88 ± 0.17b	0.59 ± 0.03b
		4C2S	8.82 ± 0.09a	8.69 ± 0.25b	0.59 ± 0.02a	5.98 ± 0.74c	1.04 ± 0.004a
		4C4S	8.83 ± 0.06a	8.02 ± 0.24c	0.56 ± 0.12a	8.53 ± 0.74b	1.10 ± 0.02a
	Soybean	SJSJ	8.75 ± 0.01a	5.16 ± 0.22e	0.68 ± 0.11b	6.66 ± 0.59b	0.32 ± 0.03e
		2C2S	8.67 ± 0.06a	10.70 ± 0.88b	0.65 ± 0.03b	7.86 ± 0.61ab	0.65 ± 0.01b
		2C4S	8.59 ± 0.14a	9.03 ± 0.43c	0.82 ± 0.01a	8.89 ± 1.462a	0.52 ± 0.01d
		4C2S	8.51 ± 0.02a	6.45 ± 1.45d	0.38 ± 0.01c	9.06 ± 0.44a	0.97 ± 0.003a
		4C4S	8.55 ± 0.01a	13.71 ± 0.31a	0.41 ± 0.03c	4.26 ± 0.16c	0.60 ± 0.01c

Different small letters indicate significant difference at *P*<0.05. The same as below.

In the soybean system, the activities of urease and sucrase in all intercropping treatments were significantly higher than those in the SJSJ monoculture. Specifically, urease activity increased by 25.00%-165.70%, and sucrase activity increased by 62.50%-203.13% (*P* < 0.05). The alkaline phosphatase activity of the 2C4S treatment was significantly higher than that of other treatments, with increases of 20.59%, 26.15%, 115.79%, and 100.00% compared with the SJSJ monoculture, 2C2S, 4C2S, and 4C4S treatments, respectively (*P* < 0.05). Meanwhile, its catalase activity showed no significant differences from that of the 2C2S and 4C2S treatments (*P* > 0.05), but was significantly higher than that of the SJSJ monoculture and 4C4S treatment (*P* < 0.05). However, the sucrase activity of the 2C4S treatment was significantly lower than that of other intercropping treatments, decreasing by 20.00%, 46.39%, and 13.33% compared with the 2C2S, 4C2S, and 4C4S treatments, respectively (*P* < 0.05). This indicates that intercropping can generally enhance soil urease and sucrase activities in the soybean system, but different intercropping patterns exhibit specificity in regulating soil enzymes. The 2C4S treatment shows a distinct advantage in enhancing alkaline phosphatase activity, yet performs less effectively in promoting sucrase activity.

In **Table 2**, in the maize system for 2023, the soil pH of the 2C4S treatment exhibited no significant difference from the CJCJ monoculture (*P*>0.05), but was significantly lower than that of the 2C2S and 4C4S treatments (*P* < 0.05). For enzyme activities, the 2C4S treatment exhibited significantly higher urease, catalase, and sucrase activities than all other treatments, with respective increases of 57.8%-216.5%, 27.2%-74.1%, and 16.8%-252.3% (*P* < 0.05). Regarding alkaline phosphatase activity, it was higher than that of the 4C2S treatment yet showed no significant difference between the two (*P*>0.05), while being notably higher than that of the other treatments (*P*<0.05).

**Table 2:** Effects of different intercropping patterns of maize and soybean on soil physicochemical properties.

Year	Crop	Treatments	pH	Urease activity (U·g^-1^)	Alkaline phosphatase activity (U·g^-1^)	Catalase activity (U·g^-1^)	Sucrase activity (U·g^-1^)
2023	Maize	CJCJ	8.16 ± 0.03bc	13.28 ± 1.06c	0.90 ± 0.14b	9.21 ± 0.29b	0.67 ± 0.03d
		2C2S	8.59 ± 0.01a	9.27 ± 0.41d	0.87 ± 0.07c	10.07 ± 0.94b	0.65 ± 0.03d
		2C4S	8.12 ± 0.03c	29.34 ± 1.01a	1.24 ± 0.02a	12.81 ± 0.51a	2.29 ± 0.001a
		4C2S	7.96 ± 0.03d	18.59 ± 0.88b	1.07 ± 0.09ab	7.36 ± 0.32c	1.36 ± 0.002c
		4C4S	8.23 ± 0.03b	9.51 ± 0.62d	0.36 ± 0.01d	9.39 ± 0.44b	1.96 ± 0.01b
	Soybean	SJSJ	8.63 ± 0.01a	9.12 ± 1.03d	0.73 ± 0.039b	9.05 ± 0.33b	0.67 ± 0.05d
		2C2S	8.60 ± 0.01a	13.76 ± 0.75c	0.54 ± 0.03c	11.45 ± 0.68a	0.96 ± 0.01c
		2C4S	8.25 ± 0.01b	21.55 ± 1.13b	0.90 ± 0.07a	9.48 ± 0.25b	1.56 ± 0.04b
		4C2S	7.96 ± 0.03c	33.93 ± 1.73a	0.98 ± 0.02a	12.12 ± 0.17a	1.95 ± 0.05a
		4C4S	8.23 ± 0.03b	12.71 ± 1.65cd	0.61 ± 0.02bc	6.83 ± 0.94c	0.38 ± 0.038e

CJCJ: Maize monoculture. SJSJ: Soybean monoculture. 2C2S: 2 rows of maize intercropped with 2 rows of soybean. 2C4S: 2 rows of maize intercropped with 4 rows of soybean. 4C2S: 4 rows of maize intercropped with 2 rows of soybean. 4C4S: 4 rows of maize intercropped with 4 rows of soybean. The lowercase letters in the tables indicate statistical differences among treatments: different lowercase letters represent significant differences at the *P*<0.05 level.

In the soybean system, the urease and sucrase activities of the 4C2S treatment were significantly higher than those of other treatments, with sequential increases of 57.4%-272.0% and 25.0%-413.2%, respectively (*P* < 0.05). Its alkaline phosphatase activity was not significantly different from that of the 2C4S treatment (*P*>0.05), but was significantly higher than that of the other treatments (*P* < 0.05). As for catalase activity, it exhibited no significant difference from that of the 2C2S treatment (*P*>0.05), whereas it was significantly higher than that of the other treatments (*P* < 0.05).

Overall, results from **Tables 1** and **2** show that the 2C4S intercropping treatment significantly promotes the activities of four key soil enzymes in maize and legume systems, with slight variations across years and enzyme types. In maize, 2C4S consistently increases soil pH and enhances urease, alkaline phosphatase, and sucrase activities. In legumes, it exhibits a distinct advantage in promoting alkaline phosphatase but performs poorly in sucrase promotion. The 4C2S treatment shows a significant effect in the 2023 soybean system. These regulations have ecological significance: ammonium nitrogen produced by urease-mediated urea decomposition in the soybean rhizosphere can supply maize, and part of the nitrogen absorbed by maize comes from nitrogen fixation by soybean root nodules.

### 3.3 Effects of different intercropping patterns of maize and soybean on crop root morphology

Based on the measurements of maize root traits at the V6 and V12 stages during 2022-2023 (**Table 3**), with the monoculture CJCJ as the control, the regulatory effects of intercropping treatments on root growth are summarized as follows:

**Table 3:** Effects of different intercropping patterns on maize root morphology.

Year	Growth stage	Treatment	Root dry weigh (g·plant^-1^)	Root length (cm·plant^-1^)	Root surface area (cm^2^·plant^-1^)	Average root diameter (mm·plant^-1^)	Root volume (cm^3^·plant^-1^)
2022	V6	CJCJ	1.29 ± 0.18b	794.80 ± 38.33c	167.22 ± 3.66b	2.30 ± 0.18a	3.21 ± 0.25a
		2C2S	1.43 ± 0.31ab	600.35 ± 3.35d	130.61 ± 1.96d	1.75 ± 0.25b	2.06 ± 0.36a
		2C4S	1.24 ± 0.13b	844.49 ± 2.31b	154.71 ± 4.50bc	2.15 ± 0.29ab	2.71 ± 0.59ab
		4C2S	1.58 ± 0.21a	846.86 ± 15.13b	143.42 ± 5.15c	2.03 ± 0.25ab	2.41 ± 0.29b
		4C4S	1.66 ± 0.22a	1139.22 ± 10.16a	185.97 ± 6.64a	2.62 ± 0.46a	2.86 ± 0.69ab
	V12	CJCJ	5.93 ± 0.95a	3429.22 ± 76.72a	558.67 ± 34.79a	5.94 ± 0.37a	8.98 ± 0.91b
		2C2S	3.63 ± 0.09c	2050.62 ± 79.19cd	355.41 ± 36.09b	4.03 ± 0.57b	6.22 ± 1.26c
		2C4S	4.53 ± 0.32ab	1889.13 ± 50.97d	370.69 ± 17.46b	4.77 ± 0.79b	7.76 ± 0.58bc
		4C2S	4.89 ± 0.47ab	2589.42 ± 102.95b	541.50 ± 11.01a	5.95 ± 0.15a	12.17 ± 0.12a
		4C4S	2.99 ± 0.31d	2148.34 ± 75.48c	363.47 ± 12.22b	4.67 ± 0.65b	7.15 ± 0.60bc
2023	V6	CJCJ	0.66 ± 0.12ab	364.80 ± 24.48c	96.37 ± 5.04a	0.81 ± 0.07b	1.36 ± 0.12b
		2C2S	0.47 ± 0.07c	308.80 ± 19.49d	67.84 ± 4.36b	0.82 ± 0.06b	1.34 ± 0.16b
		2C4S	0.62 ± 0.05b	416.82 ± 17.83bc	105.77 ± 12.68a	0.74 ± 0.05c	2.03 ± 0.29a
		4C2S	0.79 ± 0.07a	458.03 ± 9.52ab	104.41 ± 13.86a	1.17 ± 0.08a	2.34 ± 0.23a
		4C4S	0.86 ± 0.09a	497.23 ± 17.73a	121.57 ± 6.18a	0.75 ± 0.01c	2.35 ± 0.03a
	V12	CJCJ	16.92 ± 0.38a	2171.22 ± 3.66d	374.54 ± 10.57d	11.65 ± 0.09c	13.29 ± 0.29bc
		2C2S	6.38 ± 0.88c	2966.82 ± 107.23c	688.65 ± 7.40c	12.73 ± 0.35bc	15.25 ± 0.38a
		2C4S	12.35 ± 0.61b	4310.83 ± 381.73a	938.22 ± 97.26b	19.33 ± 0.55a	12.54 ± 0.63c
		4C2S	12.30 ± 1.53b	1456.77 ± 167.03e	237.14 ± 11.81e	13.43 ± 0.41b	8.74 ± 0.32d
		4C4S	11.27 ± 0.64b	3480.23 ± 182.53b	1177.82 ± 32.17a	8.82 ± 0.85d	14.06 ± 0.51ab

CJCJ: Maize monoculture. SJSJ: Soybean monoculture. 2C2S: 2 rows of maize intercropped with 2 rows of soybean. 2C4S: 2 rows of maize intercropped with 4 rows of soybean. 4C2S: 4 rows of maize intercropped with 2 rows of soybean. 4C4S: 4 rows of maize intercropped with 4 rows of soybean. The lowercase letters in the tables indicate statistical differences among treatments: different lowercase letters represent significant differences at the *P*<0.05 level.

At the V6 stage in 2022, the root dry weights of the 4C2S and 4C4S treatments were significantly higher than that of CJCJ by 22.5% and 28.7%, respectively (*P<* 0.05). Notably, the 4C2S treatment further demonstrated superiority in root length and root surface area—both of which were significantly higher than those of CJCJ by 43.3% and 11.2%, respectively (*P<* 0.05). At the V12 stage, the root volume of the 4C2S treatment was significantly higher than that of CJCJ by 35.5% (*P<* 0.05), which was the highest among all treatments. By contrast, the root dry weights of the 2C2S and 4C4S treatments were significantly lower than that of CJCJ by 38.8% and 49.6%, respectively (*P<* 0.05), indicating that interspecific competition inhibited root dry matter accumulation under certain intercropping patterns.

At the V6 stage in 2023, the 4C2S and 4C4S treatments consistently promoted root dry weight, resulting in significant increases of 19.7% and 30.3% relative to CJCJ, respectively (*P<* 0.05). Additionally, the average root diameter of the 4C2S treatment was significantly greater than that of CJCJ by 44.4% (*P<* 0.05), and the root volumes of the 2C4S, 4C2S, and 4C4S treatments were significantly higher than that of CJCJ by 49.3%, 72.1%, and 72.8%, respectively (*P<* 0.05). Upon entering the V12 stage, the 2C2S treatment exhibited remarkable root expansion capacity, with its root length and average root diameter being significantly higher than those of CJCJ by 98.5% and 65.9%, respectively (*P<* 0.05); the root surface area of the 4C4S treatment was significantly higher than that of CJCJ by 214.5% (*P<* 0.05), the highest among all treatments; while the root volume of the 2C2S treatment was also significantly higher than that of CJCJ by 14.8% (*P<* 0.05).

Cross-annual analysis revealed that the 4C2S and 4C4S treatments stably promoted root dry weight at the V6 stage. Whereas the promoting effects of intercropping treatments on root spatial expansion at the V12 stage displayed interannual variability, they were generally superior to the monoculture. Furthermore, 4C2S facilitated targeted root thickening at the V6 stage, while 2C2S enhanced root volume at the V12 stage. These two treatments thus provided a significant physiological foundation for enhancing the nutrient absorption and anchoring capabilities of maize roots—through root morphological optimization and functional capacity enhancement, respectively.

Based on the measurements of soybean root traits at the V1 and R1 during 2022-2023 (**Table 4**), with monoculture SJSJ as the control, the regulatory impacts of intercropping treatments were analyzed.

**Table 4:** Effects of different intercropping patterns on root morphology of soybean.

Year	Growth stage	Treatment	Root dry weigh (g plant^-1^)	Root length (cm plant^-1^)	Root surface area (cm^2^ plant^-1^)	Average root diameter (mm plant^-1^)	Root volume (cm^3^ plant^-1^)
2022	V1	SJSJ	0.28 ± 0.04a	188.13 ± 11.88b	18.17 ± 1.21b	0.45 ± 0.01a	0.31 ± 0.01a
		2C2S	0.24 ± 0.05b	209.91 ± 10.02a	24.16 ± 1.20a	0.47 ± 0.01a	0.29 ± 0.01a
		2C4S	0.19 ± 0.03c	162.50 ± 14.95c	19.91 ± 1.18b	0.40 ± 0.02b	0.20 ± 0.01c
		4C2S	0.24 ± 0.06b	209.96 ± 10.02a	18.49 ± 1.58b	0.34 ± 0.02c	0.20 ± 0.01c
		4C4S	0.27 ± 0.02a	210.96 ± 27.28a	25.65 ± 1.75a	0.40 ± 0.03b	0.25 ± 0.01b
	R1	SJSJ	0.88 ± 0.16a	236.86 ± 3.75c	46.00 ± 9.19a	0.97 ± 0.15b	1.02 ± 0.09a
		2C2S	0.60 ± 0.04b	372.61 ± 46.41ab	59.71 ± 10.95a	0.92 ± 0.14c	0.90 ± 0.21ab
		2C4S	0.89 ± 0.12a	436.95 ± 24.59a	69.77 ± 6.25a	1.20 ± 0.15a	1.02 ± 0.12a
		4C2S	0.53 ± 0.09b	291.17 ± 34.37b	49.12 ± 2.04a	0.63 ± 0.27e	0.44 ± 0.07c
		4C4S	0.76 ± 0.02ab	265.28 ± 3.62b	52.94 ± 9.26a	0.75 ± 0.11de	0.53 ± 0.06c
2023	V1	SJSJ	0.23 ± 0.02a	97.57 ± 0.77c	17.79 ± 2.76b	0.54 ± 0.04b	0.24 ± 0.03b
		2C2S	0.25 ± 0.03a	165.41 ± 0.88a	24.73 ± 2.40a	0.48 ± 0.03c	0.30 ± 0.04a
		2C4S	0.23 ± 0.02a	78.52 ± 3.99d	16.08 ± 1.17b	0.67 ± 0.04a	0.27 ± 0.02ab
		4C2S	0.21 ± 0.02a	105.66 ± 1.91c	17.73 ± 1.05b	0.56 ± 0.06b	0.24 ± 0.03b
		4C4S	0.16 ± 0.02b	145.99 ± 4.14b	15.88 ± 1.91b	0.35 ± 0.01d	0.14 ± 0.02c
	R1	SJSJ	10.04 ± 7.20a	338.63 ± 49.37ab	85.55 ± 10.95a	1.30 ± 0.13b	1.98 ± 0.33ab
		2C2S	2.64 ± 0.36b	462.37 ± 34.31a	87.24 ± 2.91a	1.31 ± 0.06b	1.64 ± 0.23b
		2C4S	2.67 ± 0.06b	321.87 ± 50.04ab	91.75 ± 6.64a	1.42 ± 0.13b	2.24 ± 0.20a
		4C2S	2.81 ± 0.26b	280.83 ± 27.53b	77.16 ± 6.25a	1.89 ± 0.05a	2.28 ± 0.05a
		4C4S	2.23 ± 0.72c	321.85 ± 50.19ab	77.06 ± 5.09a	1.12 ± 0.22b	1.07 ± 0.10c

CJCJ: Maize monoculture. SJSJ: Soybean monoculture. 2C2S: 2 rows of maize intercropped with 2 rows of soybean. 2C4S: 2 rows of maize intercropped with 4 rows of soybean. 4C2S: 4 rows of maize intercropped with 2 rows of soybean. 4C4S: 4 rows of maize intercropped with 4 rows of soybean. The lowercase letters in the tables indicate statistical differences among treatments: different lowercase letters represent significant differences at the *P*<0.05 level.

For the V1 stage in 2022, the root lengths of the 2C2S, 4C2S, and 4C4S treatments exhibited a significant increase of 11.6%-12.1% compared to SJSJ (*P* < 0.05). Notably, the 2C2S and 4C4S treatments further drove a synchronous and significant elevation in root surface area, ranging from 32.9% to 41.2% relative to SJSJ (*P* < 0.05). By comparison, the root dry weights of the 2C2S and 4C2S treatments were significantly reduced by 14.3%-32.1% (*P* < 0.05), and their root volumes also declined notably by 35.5% (*P* < 0.05). Upon entering the R1 stage, the 2C4S treatment stood out most prominently: not only did its root length increase significantly by 84.5% compared to SJSJ (*P* < 0.05), but its average root diameter also saw a synchronous significant rise of 23.7% (*P* < 0.05). The 2C2S treatment similarly achieved a significant 57.3% increase in root length (*P* < 0.05), yet the root dry weights of both 2C2S and 4C2S remained significantly reduced (*P<* 0.05), with their root volumes dropping even more sharply by 48.0%-56.9% (*P* < 0.05).

At the V1 stage in 2023, the 2C2S treatment maintained stable promotion of root spatial expansion: its root length was significantly 69.5% longer than that of SJSJ (*P* < 0.05), while its root surface area and root volume were also significantly enhanced by 38.9% and 25.0%, respectively (*P* < 0.05). The 2C4S treatment, by contrast, focused on optimizing root morphology, with its average root diameter showing a significant 24.1% increase compared to the control (*P* < 0.05). In sharp contrast, multiple traits of the 4C4S treatment were inhibited: its root length, average root diameter, and root volume were significantly lower than the control by 19.5%, 35.2%, and 41.7%, respectively (*P* < 0.05). By the R1 stage, all intercropping treatments displayed a significant reduction in root dry weight relative to SJSJ, with the decline ranging from 72.0% to 77.8% (*P* < 0.05). Even so, the 2C2S treatment still significantly boosted root length by 36.5% (*P* < 0.05), while the 4C2S and 2C4S treatments enhanced root storage capacity by increasing root volume (*P<* 0.05). Among these, the 4C2S treatment additionally achieved a significant 45.4% increase in average root diameter (*P* < 0.05).

Cross-annual comprehensive analysis revealed that the 2C2S treatment showed consistent promotion of root length and root surface area in early-stage soybeans. While intercropping generally exerted an inhibitory effect on root dry weight at the R1 stage, 4C2S could adapt to the intercropping setting by regulating root thickness, and 2C4S by optimizing root volume—thereby providing differentiated physiological support for improving the nutrient absorption capacity of soybean roots.

### 3.4 Effects of different maize-soybean intercropping patterns on maize and soybean yield

**Figure 5:**
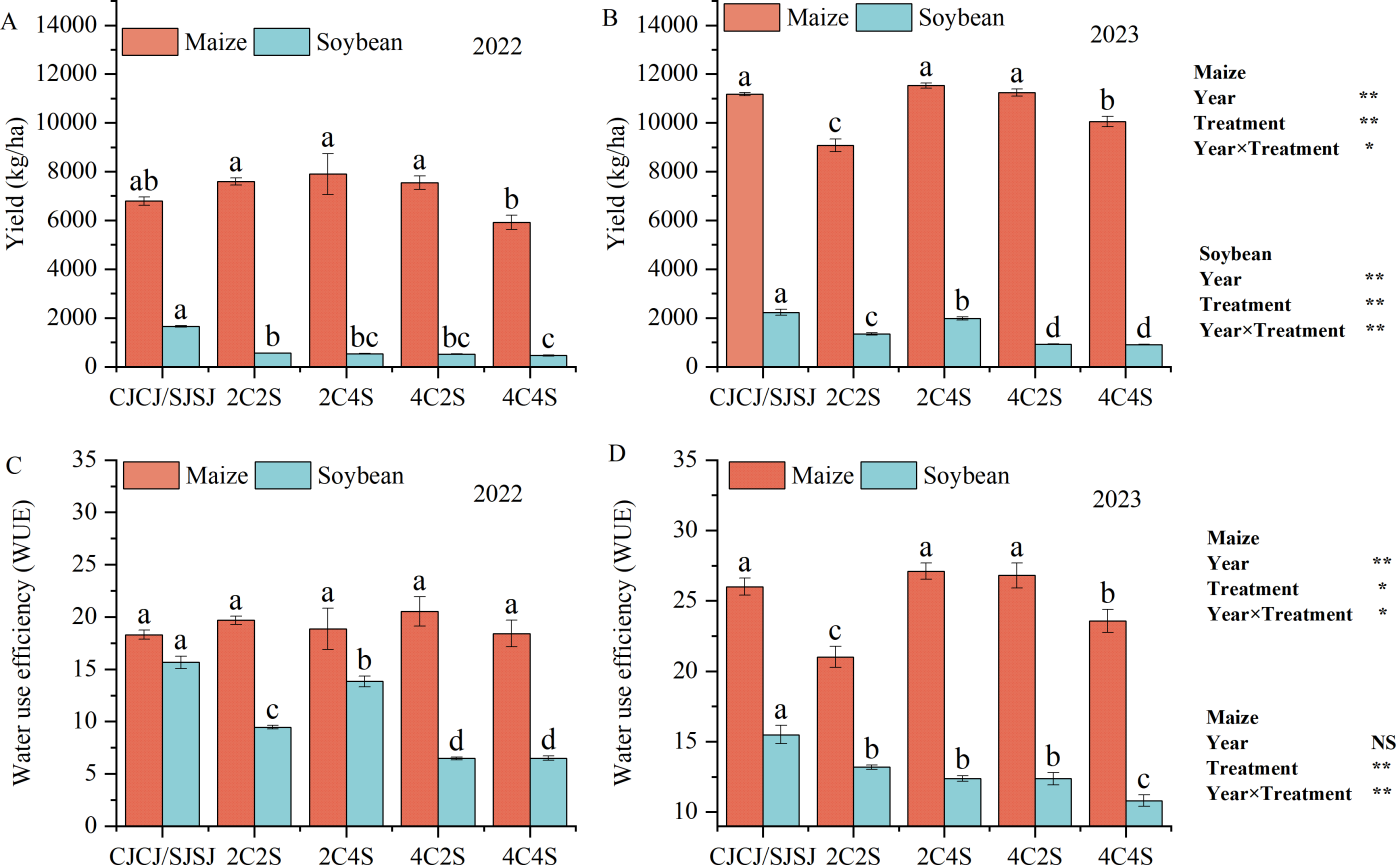
Effects of different intercropping patterns on yields and water use efficiency (WUE) of maize and soybean. Values are presented as means, and error bars indicate standard deviations. Different lowercase letters above bars or data points denote significant differences among treatments at the *P* < 0.05 level. The same below. **(A)** Maize yield in 2022; **(B)** Soybean yield in 2023; **(C)** Maize water use efficiency in 2022; **(D)** Soybean water use efficiency in 2023. *Significant difference at the 0.05 level; **Extremely significant difference at the 0.01 level; NS, No significant difference.

Significant interannual variation was observed in crop yield and WUE under film-edge cultivation across different intercropping patterns (**Figure 5**). Moreover, the Year×Treatment interaction was significant for maize yield and water use efficiency (WUE) (*P* < 0.05), and highly significant for soybean yield and WUE (*P* < 0.01). This indicates that while crop responses to intercropping fluctuated with annual environmental variability, management practices exerted predictable and dynamic effects on system performance—laying a foundation for resilient, long-term production.

In 2022, maize yield followed the descending order: 2C4S > 2C2S > 4C2S > CJCJ > 4C4S, with 2C4S achieving the highest yield. However, intercropping had no significant effect on maize WUE. Soybean yield in all intercropping treatments was significantly lower than in the monoculture SJSJ (*P* < 0.05), with the 2C2S treatment showing the highest yield among intercropping patterns. Notably, soybean WUE in the 2C4S treatment was significantly elevated by 27.97% to 47.12% compared to other intercropping treatments (*P* < 0.05).

In 2023, maize yield under 2C4S surpassed that of CJCJ by 3.19%, and its WUE was significantly higher than all other treatments by 1.14%–22.47% (*P* < 0.05). Although the soybean yield in 2C4S was 11.51% lower than SJSJ, it still outperformed other intercropping patterns. However, soybean WUE in all intercropping treatments was significantly reduced compared to monoculture, with declines ranging from 14.94% to 30.28% (*P* < 0.05).

Despite the significant Year×Treatment interactions, the 2C4S intercropping pattern consistently enhanced maize yield and WUE across both years, a trait critical for sustainable agriculture—stable improvements in the main crop’s (maize) productivity and resource use efficiency across variable climatic conditions reduce dependence on annual environmental unpredictability. While soybean yield and WUE faced constraints from interspecific competition, the system-level benefits of 2C4S (e.g., optimized land use, complementary resource acquisition between crops) underscore its potential for long-term, resilient agroecosystem management. These findings provide valuable guidance for balancing yield and resource utilization in film-edge intercropping systems to achieve sustainable production.

### 3.5 Changes in rhizosphere soil microbial diversity under different maize-soybean intercropping patterns

#### 3.5.1 Changes in soil microbial α-diversity

**Figure 6:**
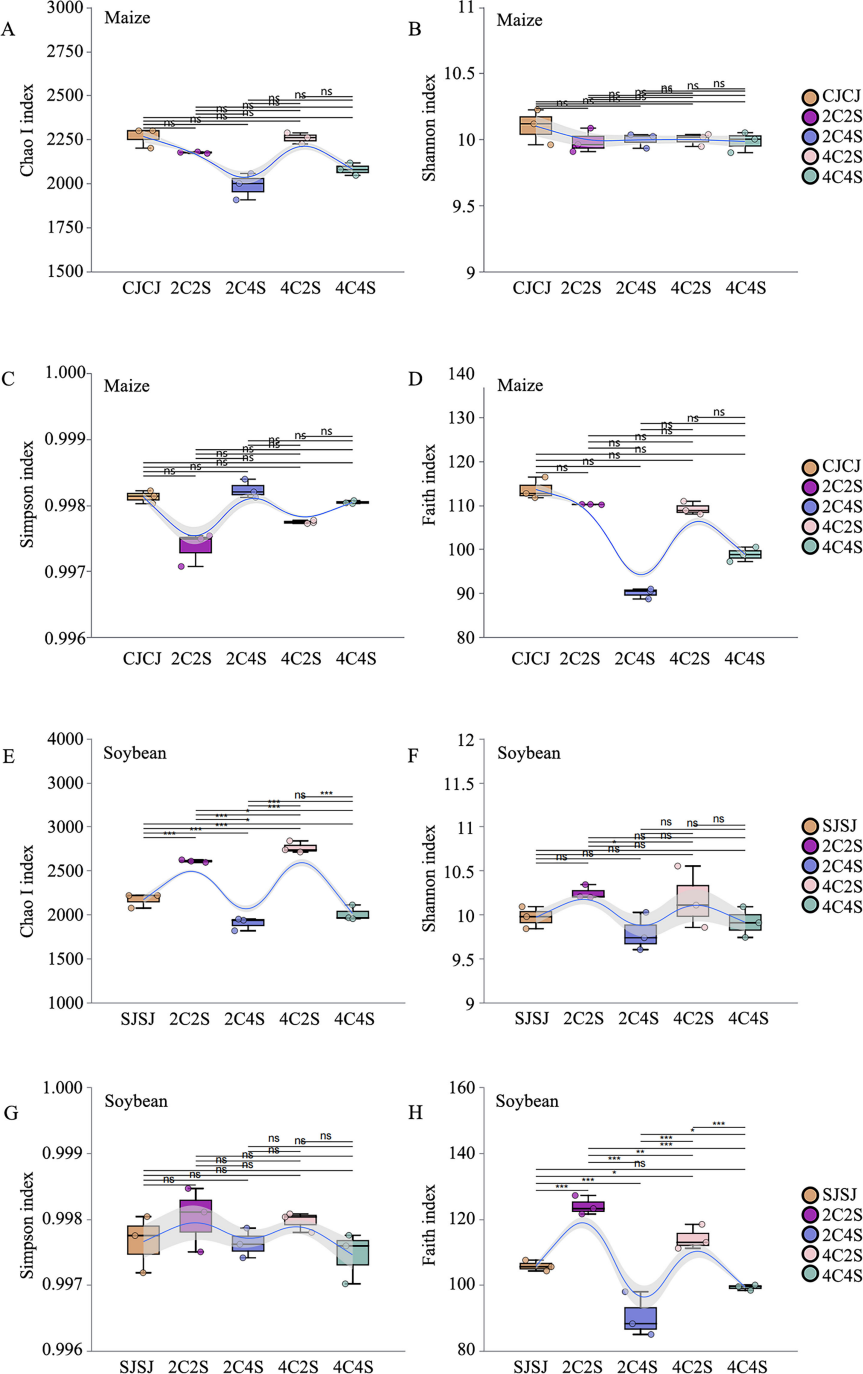
Effects of different intercropping patterns on soil microbial α-diversity. **(E–H)** show soybean data for Chao, Shannon, Simpson, and Faith indices. *: *P*<0.05; **: *P*<0.01; ***: *P*<0.001; ns: No significant difference.

The coverage depth index of all processed samples exceeded 0.99, indicating that the sequencing results accurately captured the microbial communities present in each soil sample (**Figure 6**). Chao1 and ACE indices reflect species richness, while the Shannon and Simpson indices characterize community diversity. Higher values of Chao1 and ACE suggest a greater number of species, whereas elevated Shannon and Simpson indices indicate increased ecological diversity within the microbial community.

As shown in **Table 1**, both microbial abundance and diversity varied between monoculture and intercropping treatments in maize and soybean rhizospheres. In maize, the CJCJ monoculture exhibited higher Chao1, ACE, and Shannon indices than all intercropping treatments. Among which, the Simpson index showed a slight increase in the 2C4S treatment compared to the SJSJ monoculture.

In soybean, the Chao1, ACE, and Shannon indices in the 2C2S and 4C2S intercropping treatments were higher than those in the SJSJ monoculture, but had minimal effect on the Simpson index. These results suggest that intercropping did not significantly affect the species richness (Chao1 and ACE) of maize rhizosphere microbiota, whereas 2C2S and 4C2S treatments influenced the richness and diversity of microbial communities in soybean rhizospheres.

#### 3.5.2 Changes in soil microbial β-diversity

**Figure 7:**
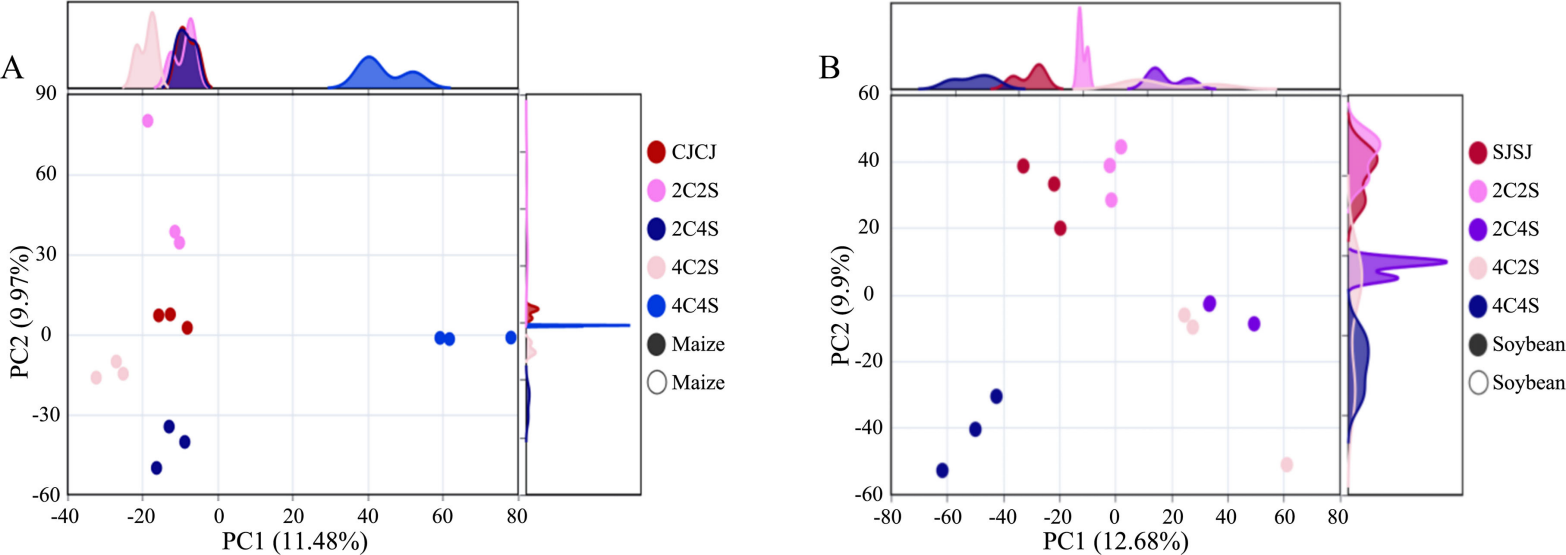
Effects of different intercropping patterns on crop rhizosphere soil microbial β-diversity. **(A)** Response of maize rhizosphere soil microbial β-diversity to different intercropping patterns; **(B)** Response of soybean rhizosphere soil microbial β-diversity to different intercropping patterns.

PCoA based on Weighted UniFrac distances was used to assess differences in rhizosphere bacterial communities across treatments (**Figure 7**). The coordinate axes with the highest contribution ratio were selected to illustrate spatial patterns. In maize rhizospheres, the first and second principal components explained 11.48% and 9.97% of the variation in community composition, respectively. All maize treatments, except 4C4S, formed closely clustered groups, indicating clear discrimination between microbial communities across treatments.

In soybean rhizospheres, the first and second principal components accounted for 12.68% and 9.90% of the variance. The sample clusters from 4C2S and 2C4S partially overlapped but remained distinguishable from other groups, demonstrating a satisfactory level of separation. These results suggest that compared with monoculture, intercropping caused more significant changes in the structure of bacterial communities, particularly in soybean, thereby indicating that cropping patterns can influence the composition of rhizosphere microbial communities.

#### 3.5.3 Effects of different maize-soybean intercropping patterns on rhizosphere soil microbial community composition

**Figure 8:**
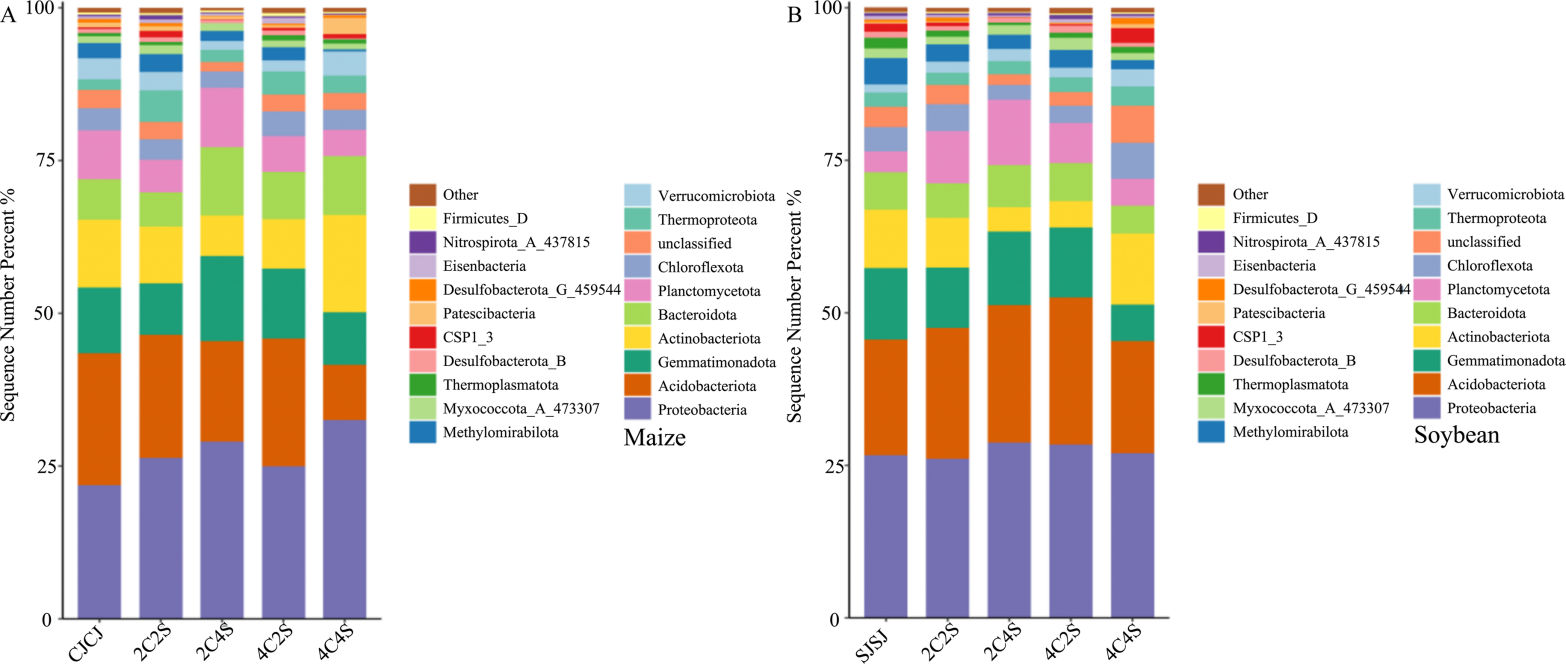
Average relative abundance of the top 20 soil bacterial populations. **(A)** Average relative abundance of the top 20 bacterial populations in maize soil; **(B)** Average relative abundance of the top 20 bacterial populations in soybean soil.

**Figure 8** illustrates the dominant bacterial phyla (top 20 by relative abundance plus others) in maize rhizosphere soils under different intercropping treatments. Community structures across treatments remained broadly consistent. Among them, the dominant bacterial phylum in each treatment was Proteobacteria, with a relative abundance of 21.79% in the CJCJ monoculture. Intercropping treatments increased the abundance of Proteobacteria by 17.02%, 33.33%, 11.29%, and 52.38%, respectively, relative to the CJCJ control. Proteobacteria have a high abundance in the rhizosphere of both maize and soybeans. These bacteria are metabolically active, and some strains can secrete extracellular polymeric substances, which can construct a microenvironment between the roots of the two crops, facilitating the transfer of nutrient molecules. The average relative abundance of Acidobacteriota across treatments was 23.81%, 20.21%, 16.44%, 20.95%, and 0.90%, respectively, while 2C4S markedly increased the abundance of Gemmatimonadota by 29.91% over CJCJ.

In soybean rhizospheres, a similar analysis revealed consistent community structure across treatments, with Proteobacteria, Acidobacteriota, Gemmatimonadota, and Actinobacteria being dominant (26.61%, 18.99%, 11.66%, and 9.60% in the SJSJ monoculture, respectively). Intercropping increased the abundance of Proteobacteria by 7.89%, 6.39%, and 1.28% in the 2C4S, 4C2S, and 4C4S treatments, respectively, compared to SJSJ. Acidobacteriota increased by 13.23%, 19.05%, and 33.33% in the 2C2S, 2C4S, and 4C2S treatments, respectively. The relative abundance of Gemmatimonadota in 2C4S was 9.09% higher than in SJSJ.

**Figure 9:**
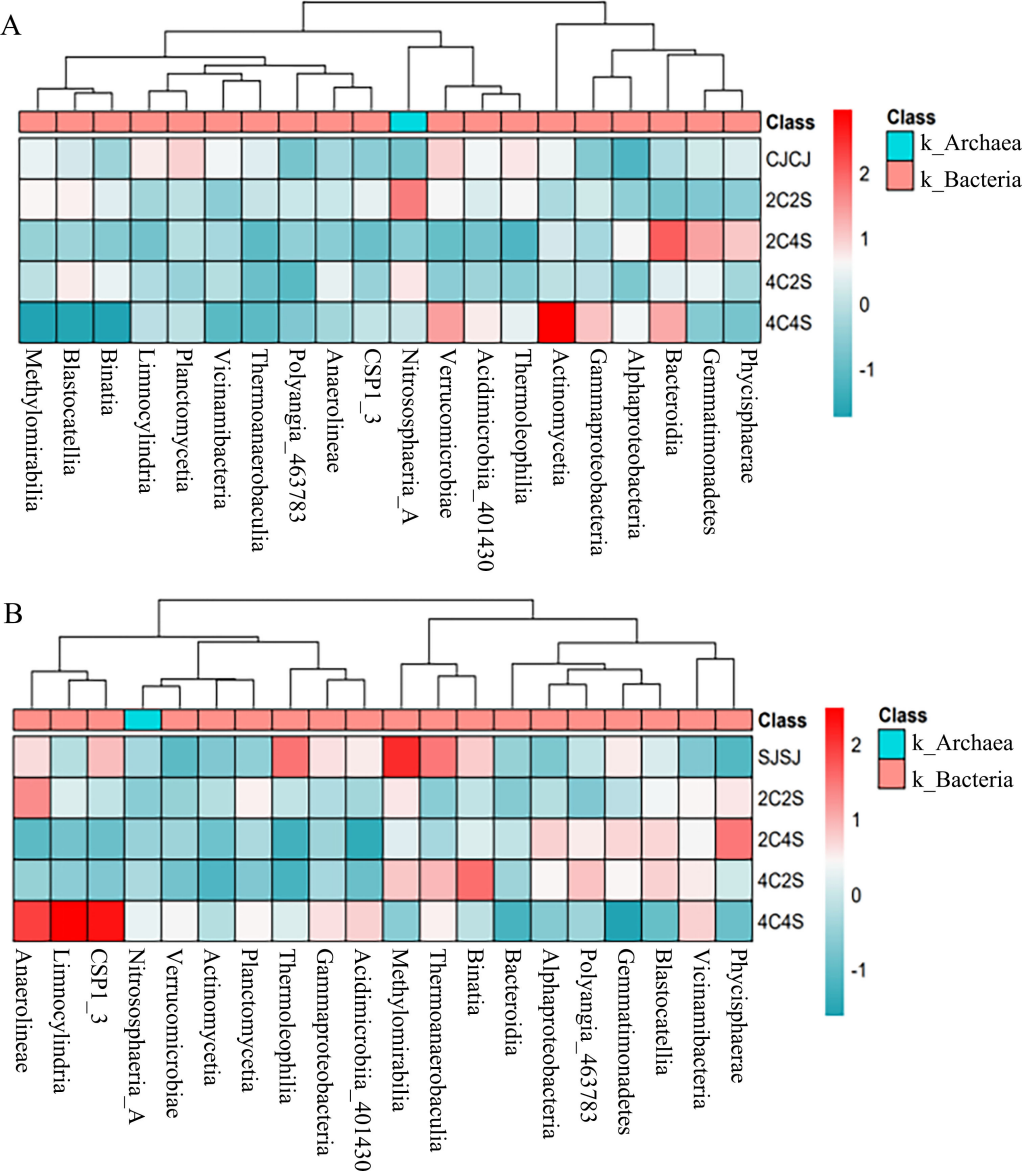
Heatmap of relative abundances of the top 20 soil bacterial taxa. **(A)** Maize soil; **(B)** Soybean soil.

**Figure 9** shows the top five most abundant bacterial classes in maize rhizospheres: Actinomycetia, Bacteroidia, Verrucomicrobiae, Nitrososphaeria-A, and Gemmatimonadetes. The 4C4S treatment yielded the highest cumulative relative abundance (52.24%) of these classes, followed by 2C4S (21.47%) and 4C2S (10.87%). Class-specific increases were observed for Actinomycetia in 4C2S, Nitrososphaeria-A in 2C4S, and Bacteroidia in 2C2S. However, no statistically significant differences were observed across treatments.

In soybean rhizospheres, the top five classes were Methanobacteria, Anaerolineae, Binatia, Thermoanaerobaculia, and Vicinamibacteria. The highest cumulative relative abundance was observed in 4C4S (62.55%), followed by the SJSJ monoculture (56%). Relative abundance of Alphaproteobacteria, Blastocatellia, and Nitrososphaeria-A was elevated in 4C2S, 2C4S, and 2C2S treatments, respectively. Despite these variations, intercropping had a limited impact on the relative abundance of dominant bacterial classes in crop rhizospheres.

#### 3.5.4 Effects of different maize-soybean intercropping patterns on bacterial species composition in rhizosphere soil

**Figure 10:**
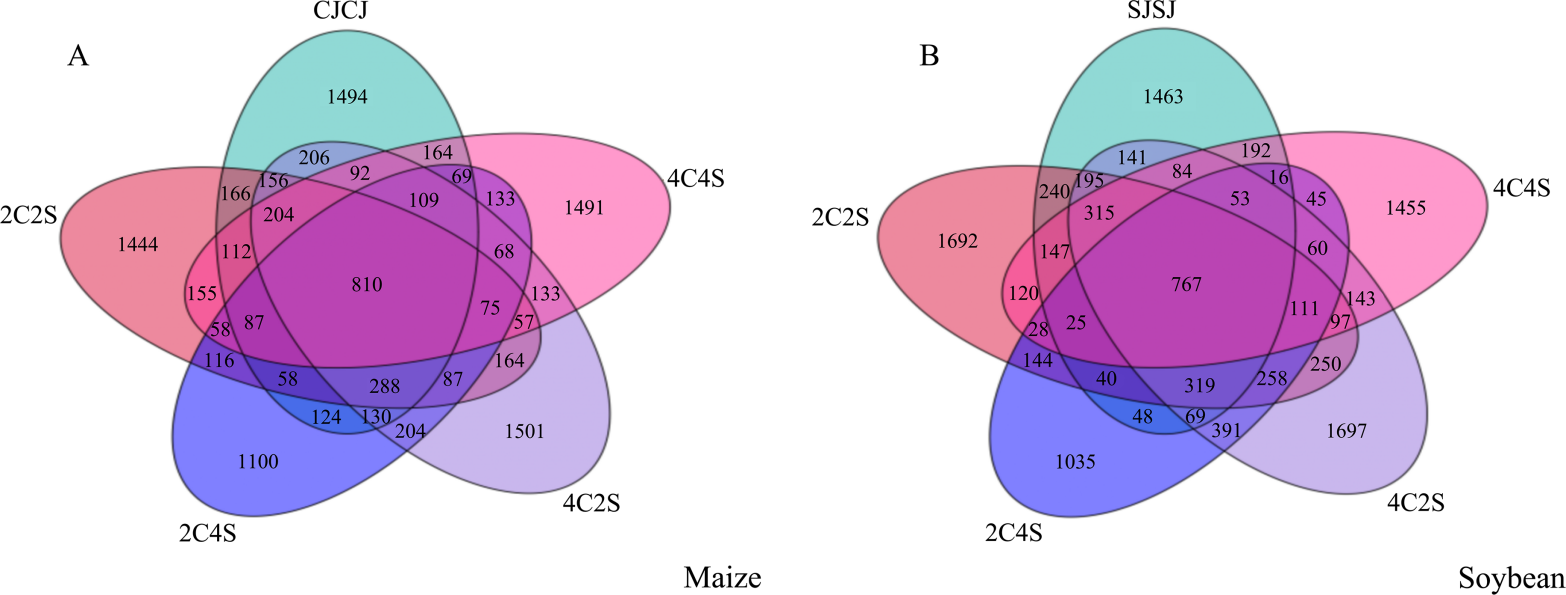
Distribution of bacterial OTUs in crop rhizosphere under different intercropping patterns. **(A)** Maize soil; **(B)** Soybean soil.

The Venn diagram provides an intuitive visualization of the overlap and uniqueness of OTU compositions among multiple samples, thereby illustrating microbial community similarity across environmental conditions. As shown in **Figure 10**, a total of 810 OTUs were identified across the five maize treatments, encompassing 51 phyla, 116 classes, 318 orders, 507 families, 911 genera, and 768 species. Among these treatments, 4C2S exhibited the highest number of unique OTUs (1501), while CJCJ, 2C2S, 2C4S, and 4C4S collectively shared 87 OTUs. Similarly, 767 OTUs were detected across the five soybean treatments. The 4C2S treatment also demonstrated the highest number of unique OTUs in the soybean system, with 1697, while SJSJ, 2C2S, 2C4S, and 4C4S shared a total of 25 OTUs.

### 3.6 Correlation between rhizosphere soil enzyme activities, microbial community composition, root traits, and crop yield

**Figure 11:**
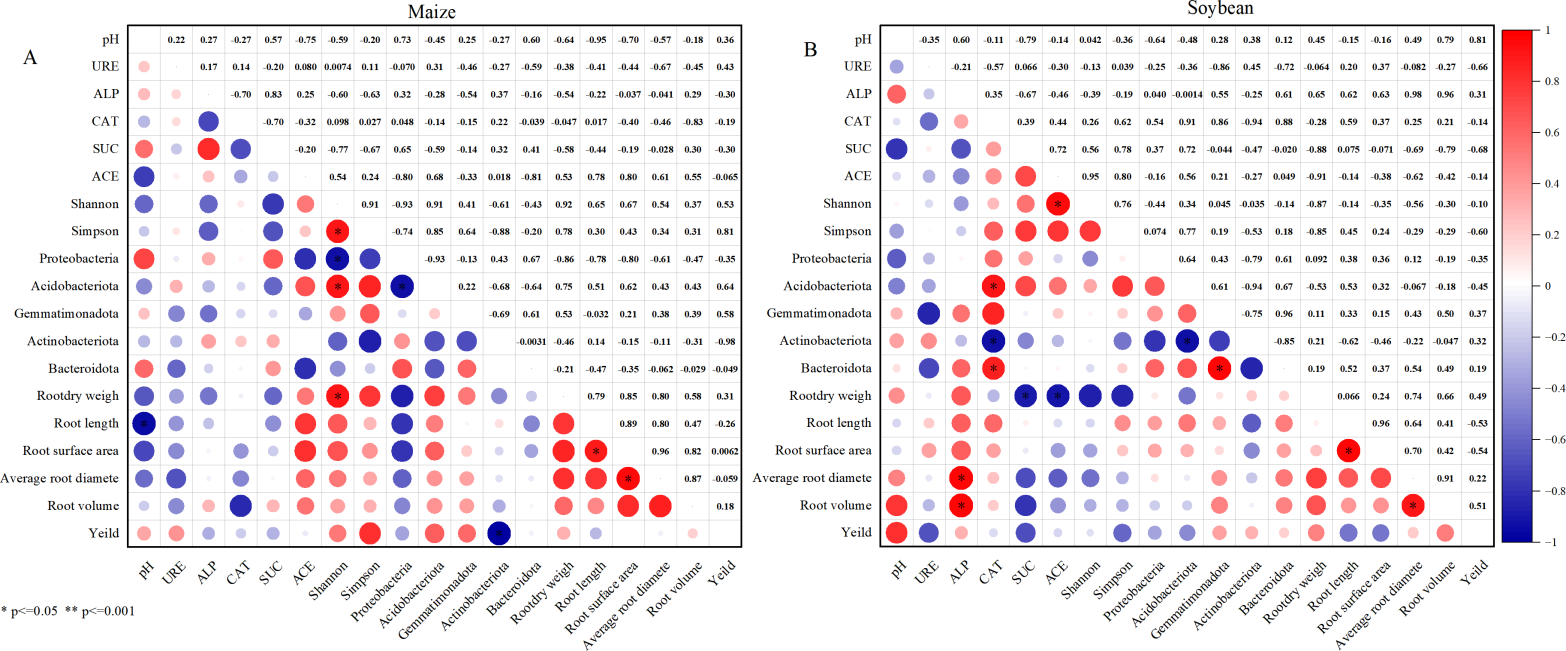
Correlation between belowground indicators and yield under different maize-soybean intercropping patterns. **(A)** Maize soil; **(B)** Soybean soil.

Correlation analysis revealed a significant negative relationship between soil pH and root length in the maize system (**Figure 11**). The ACE index was positively correlated with the root shape index, and maize yield showed significant positive associations with soil pH, as well as the relative abundances of Acidobacteriota and Gemmatimonadota. In the soybean system, yield was positively correlated with both soil pH and ALP activity. ALP activity also exhibited strong positive correlations with various root traits, particularly root volume and average root diameter, suggesting its key role in promoting root development. Additionally, CAT activity was significantly positively associated with the abundances of Bacteroidota and Acidobacteriota. Collectively, these findings indicate that dominant microbial phyla in the rhizosphere are closely linked to root trait expression and thus play an essential role in the growth and productivity of soybean under maize–soybean intercropping systems.

### 3.7 Structural equation modeling

**Figure 12:**
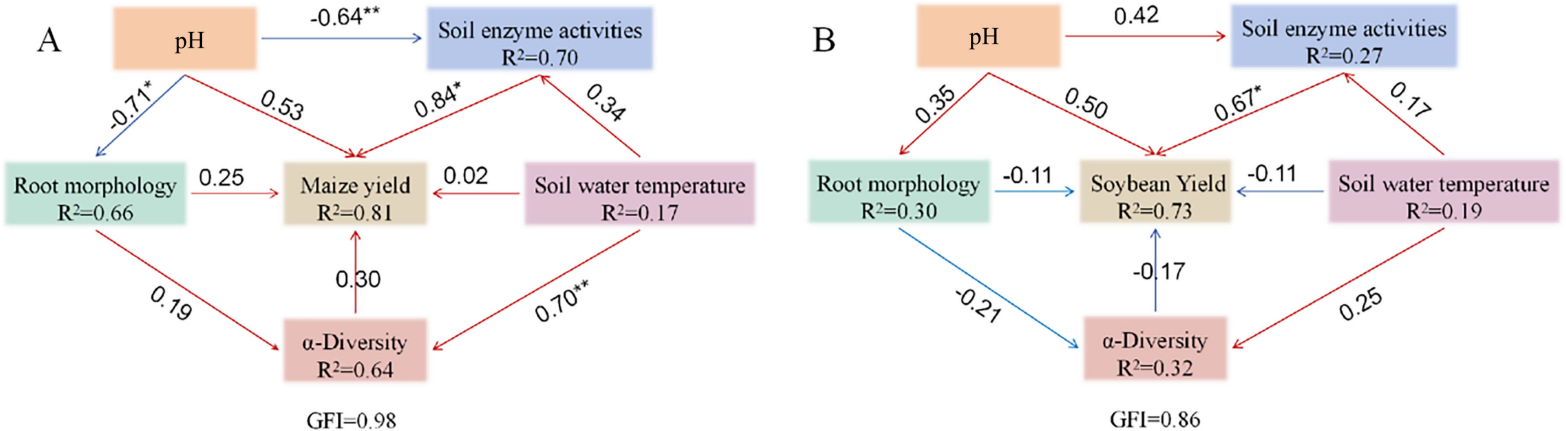
Structural equation model (SEM) illustrating the effects of soil pH, soil water and temperature, soil enzyme activities, root morphology, and microbial α-diversity on crop yield. Unidirectional arrows represent hypothesized causal directions, with values indicating path coefficients. Blue arrows denote negative effects, while red arrows denote positive effects. R² values adjacent to parameters represent the percentage of variance explained by other variables. *P < 0.05; **P < 0.01; ***P < 0.001. Models with different structures were evaluated using goodness-of-fit statistics, which measure overall predictive performance. **(A)** Maize; **(B)** Soybean.

To elucidate the interactions among key variables influencing crop yield, a structural equation model was constructed for the maize–soybean intercropping system (**Figure 12**). In the maize yield model, root morphology emerged as a critical determinant of yield, exerting a significant positive effect on maize yield (coefficient = 1.09, *P* = 0.01; standardized estimate = 0.84). Soil pH, Alpha diversity, soil enzyme activity, and soil water temperature had no direct significant effects on maize yield. However, soil water temperature has a substantial impact on Alpha diversity (coefficient = 0.80, *P* < 0.01; standardized estimate = 0.70), while enzyme activity remained unaffected.

Soil enzyme activity was influenced by root morphology, soil water temperature, and pH, with pH exhibiting a significant negative effect (*P* = 0.03). Root morphology was significantly negatively impacted by pH (*P* < 0.01) and showed an association with soil water temperature. Soil water temperature, in turn, was influenced solely by pH. Both root morphology and Alpha diversity positively influenced soil enzyme activity but negatively impacted pH, with root morphology contributing more strongly to enzyme activity than Alpha diversity. Importantly, both root morphology and soil enzyme activity had positive effects on maize yield, with the influence of enzyme activity being more pronounced. Maize yield negatively affected Alpha diversity but positively influenced pH, while Alpha diversity itself negatively regulated pH. Overall, modifications in root morphology significantly influenced the maize subsystem and, by extension, the overall dynamics of the intercropping system.

In the soybean yield model, soil enzyme activity had the most pronounced effect on yield. It also shaped Alpha diversity in concert with root morphology, although the effect of root morphology was marginally significant (*P* = 0.06), and soil water temperature had no significant influence. Root morphology was affected by enzyme activity, soil water temperature, and pH, though none of these associations were statistically significant, including the weak positive effect of pH. Similarly, enzyme activity was modulated by pH and soil water temperature, although the effects were not significant, while soil water temperature was significantly affected by pH. These results highlight a complex network of interactions among variables, where changes in enzyme activity exert a direct impact on soybean yield and initiate cascading effects. Through its modulation of soil water temperature and pH, indirect influences on other factors ultimately shape yield outcomes. Notably, the magnitude of variable interactions differed, with soil enzyme activity emerging as the most influential factor for soybean yield, playing a pivotal role in sustaining model stability and crop productivity.

## 4 Discussion

### 4.1 Effects of different maize-soybean intercropping patterns on soil hydrothermal status of maize and soybean

As a critical interface for crop-microbe interactions, soil hydrothermal dynamics exhibit multi-dimensional regulatory characteristics in the maize-soybean intercropping system. The stratified canopy structure of tall-stature (maize) and short-stature (soybean) crops creates a gradient light distribution, enhancing the transmittance of photosynthetically active radiation (PAR). This three-dimensional structure simultaneously reduces soil temperature and regulates the field microclimate through transpiration (Ai et al., 2021). Meanwhile, in the maize-soybean intercropping system, vertical stratification—characterized by deep-rooted maize and shallow-rooted soybeans—and horizontal expansion differences—with maize roots extending toward soybean rows and soybean root growth being inhibited—form staggered water absorption zones, thereby reducing interspecific competition.

This study revealed that soil water content (SWC) presents a vertical distribution pattern of first increasing then decreasing. The 0–20 cm topsoil layer has relatively low SWC due to atmospheric evaporation and water uptake by shallow roots, while the 40–60 cm deep soil layer forms a significant water deficit zone, attributed to water consumption by deep maize roots and water infiltration obstruction by the clay layer. The intercropping system reduces evaporation in the soybean zone via maize shading; concurrently, the complementarity between deep and shallow roots alleviates water competition, maintaining soil moisture in the intercropped soybean zone within an optimal threshold. Soil temperature decreases exponentially with increasing soil depth, and differences in temperature among different treatments converge as depth increases. Maize canopy coverage enhances temperature buffering capacity, while the soybean zone forms a stable microclimate due to the shading effect.

Under the extreme climatic conditions of high temperature and low rainfall during the grain R4 stage in 2023, the 4C4S treatment (a specific row ratio treatment) intensified water competition due to high-density planting, resulting in significant interannual fluctuations in soil moisture. In contrast, the intercropping pattern with optimized row ratios enhanced system stability through structural complementarity. This row ratio-dependent hydrothermal regulation mechanism provides a structural optimization basis for constructing drought-resistant intercropping systems.

### 4.2 Effects of different maize-soybean intercropping patterns on rhizosphere soil physicochemical properties

Previous studies have demonstrated that intercropping modifies soil physicochemical properties, including the optimization of soil pH and significant enhancement of soil nutrient contents (e.g., organic matter) in the plough layer, thereby promoting plant growth (Jan et al., 2016). Furthermore, intercropping systems have been shown to increase the availability of nitrogen and phosphorus, boost soil enzyme activity, and enhance land-use efficiency and economic returns (Liu et al., 2024a). These systems often result in greater crop yields and nutrient uptake, which in turn influence microbial activity and enzymatic dynamics in the rhizosphere (Kumar et al., 2022). Previous studies have demonstrated that intercropping patterns involving leguminous and woody plants significantly influence soil phosphatase activity (Qu et al., 2024). Additionally, intercropping treatments have been shown to exhibit significantly higher levels of soil organic matter, urease activity, and sucrase activity compared to monoculture systems (Zhou et al., 2023).

This study found that the crop-specificity and interannual dynamics in the regulation of rhizospheric soil physicochemical properties by intercropping arise from the coupled effects of crop physiological characteristics, intercropping structure, and environmental factors.

The interannual fluctuation of soil pH in the maize system is significantly greater than that in the soybean system. This may stem from the deep distribution of maize’s fibrous root system and its sensitivity to water: interannual precipitation variations not only accelerate base ion leaching but also potentially alter the root system’s perception of water stress, forcing adjustments in organic acid secretion and thereby amplifying pH fluctuations. In contrast, the release of OH^-^ during nodule nitrogen fixation and secretion of weakly alkaline exudates by soybean’s taproot system may form a stable endogenous acid-base regulation mechanism, granting it stronger adaptability to climate fluctuations.

Pattern-specific advantages in enzyme activity reflect the alignment between intercropping patterns and crop demands. For maize in 2023, the 2C4S pattern maintained high enzyme activity despite pH fluctuations, possibly because its row ratio fosters shallow-layer complementarity and deep-layer separation of root systems, reducing substrate competition and stabilizing the microbial environment. The superiority of 4C2S and 4C4S in 2022 may be linked to that year’s stable climate and balanced nutrient supply-demand. In soybeans, the prominent alkaline phosphatase activity of 2C4S in 2022 might result from soybean prioritizing resource allocation to synthesize this enzyme in response to phosphorus stress. In 2023, the high urease and sucrase activities of soybean 4C2S could be attributed to increased competition from a higher maize row ratio; soybeans may enhance nutrient transformation by boosting secretion of enzyme-inducing signaling molecules and improving nodule nitrogen fixation efficiency.

Intercropping-driven urease optimization supports interspecific coordinated nitrogen utilization, potentially a long-term adaptive nutrient-sharing strategy of crops. Ammonium nitrogen transformed by soybeans is absorbed by maize via the rhizospheric overlapping zone, a process that may involve the joint regulation of nitrogen-transforming microorganisms by root exudates.

### 4.3 Changes in crop rhizosphere soil microbial diversity under different maize-soybean intercropping patterns

In crop growth, soil bacterial communities drive key biochemical processes such as oxidation, nitrification, and ammonification, thereby mediating soil organic matter decomposition and nutrient transformation (Sun et al., 2014; Maillard et al., 2019; Sun et al., 2021). This study revealed that the intercropping system exerts specific regulation on the bacterial community structure through a “crop-microbe” interaction mechanism: the maize rhizosphere is dominated by the phylum *Proteobacteria*, while the soybean rhizosphere centers on the phylum *Acidobacteria*, with significant differences in their OTU compositions. The root cause lies in the specific selection effect of crop root exudates—organic acids secreted by maize preferentially recruit nutrient-activating taxa such as *Proteobacteria*, whereas flavonoid signaling substances secreted by soybeans specifically enrich organic matter-decomposing bacteria like *Acidobacteria* (Zhang F, et al., 2023; Benmrid et al., 2024).

Under the 2C4S intercropping pattern, the relative abundance of *Proteobacteria* in the maize rhizosphere increased by 33.33%, and the genus *Odontomonas* that is a key organic matter-decomposing functional bacterium rose by 29.91%. Meanwhile, the abundance of *Acidobacteria* in the soybean rhizosphere reached 20.95%, forming a cross-species complementary nutrient cycle. In contrast, the 4C4S intercropping pattern led to a total abundance of 52.24% for classes *Nitrososphaeria_A* and *Blastocatellia* in the maize rhizosphere, which enhanced nitrogen transformation and carbon sequestration functions.

Notably, the impact of intercropping on dominant bacterial communities is constrained by niche barriers: Owing to their microenvironment adaptability evolved over long periods, *Proteobacteria* and *Acidobacteria* remain dominant in the intercropping system, with fluctuations in their relative abundance of <34%, indicating strong niche stability of the dominant taxa. This regulatory specificity stems from a dual mechanism: The rhizosphere selection effect dominates species composition, and crop genetic traits define the framework of core bacterial communities; The “microenvironment buffering effect” induced by intercropping—by stabilizing soil temperature and moisture and enhancing enzyme activity—activates rare functional bacterial taxa rather than disrupting the structure of dominant communities.

This study confirms that the regulation of microbial communities by intercropping is functionally targeted: on the basis of maintaining the crop-dominated bacterial community pattern, it optimizes rare functional taxa to improve system productivity (Jiang et al., 2024).

### 4.4 Effects of different maize-soybean intercropping patterns on root morphology and crop yield

Previous studies revealed through maize-soybean intercropping experiments that the plasticity in root distribution and root morphology exhibited by the maize-soybean intercropping pattern promotes yield enhancement in the intercropping system (Fan et al., 2025). Using pot experiments, previous research demonstrated that intercropping patterns significantly affect soybean root traits, including root volume, root dry weight, root length, and root surface area (Ma et al., 2025). Intercropping has been shown to promote superior root traits compared to monoculture, thereby enhancing microbial function, enzymatic activity, and yield (Zhang et al., 2013; Homulle et al., 2022).

This study further reveals two prominent characteristics: crop-specificity in root morphological responses and interannual adaptability of morphology-yield coupling. From the perspective of crop-specificity, maize and soybean exhibit differentiated strategies in root responses to intercropping. Maize adopts an “expansive root strategy” by implementing targeted optimization of root spatial expansion and biomass accumulation, which expands the range of water and nutrient absorption and enhances nutrient transport efficiency. This strategy perfectly matches the high resource demand of maize in the late growth stage, serving as the key physiological basis for the 2C4S pattern to stably increase maize yield over two years. In contrast, soybean displays “compensatory root adaptation”: although root dry weight is generally inhibited at the R1 stage, the 2C2S pattern maintains root length expansion, while the 4C2S pattern increases root volume. By replacing biomass increase with morphological adjustment, soybean alleviates the limitation of interspecific competition on resource acquisition and ultimately achieves relatively high yield under intercropping. This finding complements the general understanding in previous studies that intercropped roots outperform those in monocultures, suggesting that soybean relies more on the optimization of root functional morphology rather than mere biomass accumulation under intercropping.

Regarding the interannual adaptability of morphology-yield coupling, the advantage of the 2C4S pattern is particularly significant: the optimization degree of maize root morphology in this pattern consistently forms a stable coupling with yield amid interannual climate fluctuations. Even when soil environmental fluctuations occurred due to precipitation changes in 2023, the 2C4S pattern could still ensure resource absorption through adjustments in root distribution, supporting the simultaneous increase in yield and water use efficiency. In contrast, other patterns show more obvious yield fluctuations due to insufficient interannual stability of root responses. This indicates that the yield-increasing potential of an intercropping pattern depends not only on whether root morphology is optimized, but more importantly on the adaptability between root traits and interannual environments. Only when root adjustments match the rhythm of environmental resource supply can yield gains be maximized.

## 5 Conclusion

In conclusion, the 2C4S intercropping pattern specifically enriches functional microbial taxa such as Proteobacteria and Acidobacteriota, regulates the activity expression of key rhizosphere enzymes including urease and alkaline phosphatase, while shaping the root morphology of maize to adapt to deep soil water absorption and driving soybeans to acquire resources via a morphological compensation mechanism in their root systems. Furthermore, its regulation of soil hydrothermal conditions is precisely aligned with the needs of both crops, enabling the maximization of maize production potential while sustaining the ecological functions of soybeans. This pattern fully embodies the core design principle of sustainable intercropping systems, namely yield increase of dominant crops - maintenance of ecological functions of companion crops.

These findings demonstrate that the synergistic benefits of intercropping systems arise from row ratio–mediated optimization across environmental, microbial, and crop interactions. This provides a theoretical foundation for developing intercropping strategies that simultaneously promote Yields of dominant crops and ecological resilience in arid regions.

## Data Availability

The datasets presented in this study can be found in online repositories. The names of the repository/repositories and accession number(s) can be found in the article/**Supplementary Material**.
